# Variations of Clinical Target Volume Delineation for Primary Site of Nasopharyngeal Cancer Among Five Centers in China

**DOI:** 10.3389/fonc.2020.01572

**Published:** 2020-08-20

**Authors:** Shichuan Zhang, Shuang Yang, Peng Xu, Yun Xu, Guanqun Zhou, Xiaomin Ou, Runye Wu, Mei Lan, Davide Fontanarosa, Jason Dowling, Xiaoshen Wang, Shaojun Lin, Jun-Lin Yi, Ying Sun, Chaosu Hu, Jinyi Lang

**Affiliations:** ^1^Department of Radiation Oncology, Sichuan Cancer Hospital and Institute, Radiation Oncology Key Laboratory of Sichuan Province, School of Medicine, University of Electronic Science and Technology of China, Chengdu, China; ^2^Department of Oncology, The Affiliated Hospital of Southwest Medical University, Luzhou, China; ^3^Department of Oncology, People’s Hospital of Cangxi County, Guangyuan, China; ^4^Department of Radiation Oncology, Fujian Cancer Center, Fuzhou, China; ^5^Department of Radiation Oncology, School of Medicine, Sun Yat-sen University Cancer Center, State Key Laboratory of Oncology in South China, Collaborative Innovation Center for Cancer Medicine, Guangzhou, China; ^6^Department of Oncology, Shanghai Medical College, Fudan University, Shanghai, China; ^7^Department of Radiation Oncology, National Cancer Center/Cancer Hospital, Chinese Academy of Medical Sciences, Peking Union University, Beijing, China; ^8^School of Clinical Sciences, Queensland University of Technology, Brisbane, QLD, Australia; ^9^Institute of Health & Biomedical Innovation, Queensland University of Technology, Brisbane, QLD, Australia; ^10^Australian e-Health Research Centre, CSIRO, Brisbane, QLD, Australia

**Keywords:** CTV, nasopharyngeal cancer, target contouring variations, probability heat map, CT, MRI

## Abstract

**Purpose:**

The purpose of this study is to investigate the current status of clinical target volume (CTV) delineation for primary site of nasopharyngeal cancer (NPC) among five large tertiary cancer centers in China.

**Materials and Methods:**

The simulation CT and MR images of a patient with T3N2M0 NPC were sent to the centers participating. Fourteen experienced physicians contoured the targets independently, and the outlined structures were compared. The consistency and differences among these 14 CTVs are discussed.

**Results:**

Two different CTV designs were used in the centers. “One-CTV” design defines one CTV with a dose of 60 Gy, whereas “two-CTV” design has a high-risk CTV with dose of 60 Gy and a medium risk CTV with dose of 54 Gy. We found that the coverage of prophylactic area is very consistent between these two designs. The variances on the coverage of some sites were also significant among physicians, including covering cavernous sinus at un-involved side, posterior space of styloid process, and caudal border on posterior pharyngeal wall.

**Conclusions:**

Standardization is the main requirement for personalization of care; our study shows that among the 14 physicians in the five centers the coverage of prophylactic areas is in excellent agreement. Two distinct strategies on CTV design are currently being used, and multiple controversies were found, suggesting further optimization of CTV for primary site of NPC is needed.

## Introduction

Intensity modulated radiation therapy (IMRT) is the major treatment of nasopharyngeal cancer (NPC) (1). In IMRT planning, accurate target delineation is the first critical step to ensure good tumor control. However, even in tertiary centers that treat large numbers of NPC patients, inter-physician variance on gross tumor volume (GTV) and, even more importantly, clinical target volume (CTV) delineation is still significant. Moreover, among different centers, dose prescriptions for CTV are not identical (2). This may lead to different normal tissue toxicity among centers, even though their tumor control rates are comparable.

In this study, 14 experienced (over 10 years) physicians from five large cancer centers in China outlined the CTV and prescribed treatment dose for an NPC case to investigate the variations of CTV for primary site among different centers. These CTVs were also compared with CTV suggested by an international clinical consensus recently published (3). Besides these guidelines, in China there are also recommendations based on national guidelines published in 2010 (4). It is currently unclear how the two guidelines compare to each other, in particular in terms of prophylactic coverage, which is the most used approach for treatments in China. Our results provide an answer to these questions and evidence to support an improved standardization of the CTV definition; at the same time, the design differences shown by this study may contribute to a further understanding of the criticalities in the process and to a further optimization of NPC treatments.

## Materials and Methods

### Patient

This study was approved by the Ethical Committee of the Sichuan Cancer Hospital. A patient with T3N2M0, Stage III disease (AJCC 8th edition) was selected. He was a 45-year-old male. The histopathology was non-keratinized undifferentiated nasopharyngeal carcinoma. The tumor was located in the right fossa of Rosenmuller extending to the right parapharyngeal space. Part of the right pterygoid process was also involved (see Supplementary Material).

#### Imaging

A planning CT for head and neck was acquired with 3-mm-thickness section. Iodine contrast was intravenously applied to allow better visualization of tumor tissue.

A magnetic resonance imaging (MRI) scan was performed using a 1.5 T MR system. Three different sequences were used: T1 weighted fast spin-echo images, T2 weighted fast spin-echo images, and T1 contrast enhancement with fat saturation.

### Centers and Physicians Involved

Fourteen physicians with over 10-year experience in radiation oncology were involved in this study, from five different reference centers for radiation oncology in China: one from the Cancer Hospital of Chinese Academy of Medical Science (CHCAM), one from the Fudan University Shanghai Cancer Center (FUSCC), four from Fujian Cancer Hospital (FCH), three from the Sun Yat-sen University Cancer Center (SYSUCC), and five from the Sichuan Cancer Hospital (SCH).

### Target Delineation and Data Collection

The patient medical record was shared with all the participating physicians. Both CT and MR images were sent to the five centers in DICOM format. All the physicians contoured the GTV, CTV, and CTVln (CTV for lymph nodes) independently on CT/MRI fusions following their standard intra-institutional protocol. All the structures outlined were then collected and transferred onto the same CT volume for analysis. The volume of individual CTV for primary site was derived from treatment planning system (TPS). This study only focuses on the CTV for the primary site.

### Probability Heat Map Generation

Each structure in DICOM files was imported into individual 3D binary labels in the 3D Niftii file format using the Insight Toolkit^1^. The contributing binary labels were added, and the result was then divided by the number of contributing labels to generate a probability of overlap. The resulting overlap probability images were then assigned a threshold at 0.1 probability intervals (from 0.1 to 1.0) to generate 3D binary labels representing the percentage overlap between observers. These were then converted into individual DICOM-RT structures using the code from Dowling et al. (5).

The resulting probability structures were imported into Slicer3D (6) using the Slicer-RT plugin (7), and this software was used to generate the overlay images.

### Statistical Analysis

Two-tailed *t* test was used to compare volumes of CTV between two groups.

## Results

Among the five centers, the CTV designs, either for primary site or lymph nodes, show some important differences (see Table 1). For the primary site, the focus of this study, in particular the following observations can be made.

**TABLE 1:** Different prescriptions and fractionations among the five centers.

	GTV	GTVln	CTV high-risk	CTV low-risk	CTVln high-risk	CTVln low-risk
CHCAM	73.92 Gy/33f	69.96 Gy/33f	60.06 Gy/33f		60.06 Gy/33f	50.96 Gy/28f
FUSCC	70.4 Gy/32f	66 Gy/32f	60 Gy/32f		60 Gy/32f	54 Gy/32f
FCH	70.95 Gy/33f	70.95 Gy/33f	61.05 Gy/33f	54.45 Gy/33f		54.45 Gy/33f
SYSUCC	69.96 Gy/33f	68 Gy/33f	60 Gy/33f	54 Gy/33f	60 Gy/33f	54 Gy/33f
SCH	69.96 Gy/33f	69.96 Gy/33f	66 Gy/30f	60 Gy/30f		54 Gy/30f
Consensus	70 Gy equivalent	70 Gy equivalent	70 Gy equivalent	Prophylactic dose (60 Gy)	70 Gy equivalent	50–60 Gy

*Abbreviations: GTV, gross tumor volume; GTVln, gross tumor volume for lymph nodes; CTV, clinical target volume; CTVln, clinical target volume for lymph nodes; CHCAM, the Cancer Hospital of Chinese Academy of Medical Science; FUSCC, the Fudan University Shanghai Cancer Center; FCH, Fujian Cancer Hospital; SYSUCC, the Sun Yat-sen University Cancer Center; SCH, the Sichuan Cancer Hospital.*

CHCAM and FUSCC (“one-CTV group”) have only one CTV for the primary site with a 60 Gy prescription (this CTV will therefore be referred to as CTV60 from now on). FCH and SYSUCC (“two-CTV group”) have two CTVs: a high-risk CTV and a low-risk CTV, receiving 60 Gy (CTV60) and 54 Gy (CTV54, hereafter), respectively. The CTV design and dose prescription of SCH was consistent with the international consensus, with a high-risk CTV receiving definitive dose (66 Gy), and a low-risk CTV receiving a prophylactic 60 Gy dose (CTV60). Since the comparisons proposed in this paper focus on prophylactic coverage, SCH has been classified as well as one-CTV group.

**FIGURE 1:**
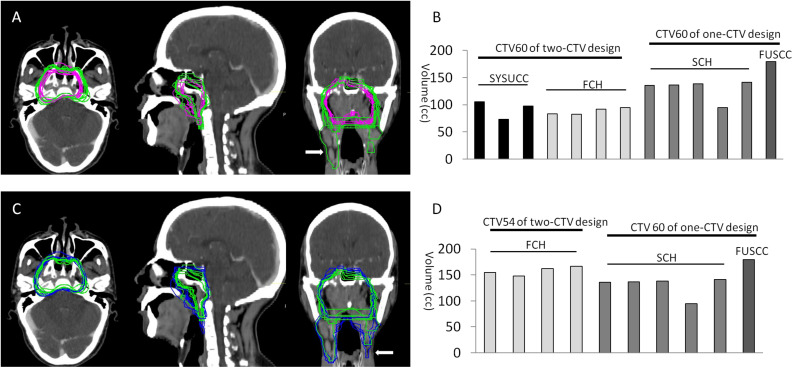
Comparison of CTV volume. **(A)** Overview of CTV60s in transverse, sagittal, and coronary sections. Note that CTV60 of CHCAM also covers part of cervical lymph node area (white arrow in coronary figure). **(B)** Comparison of volume of CTV60. The volume of CTV60 from two-CTV design group (FCH AND SYSUCC) is significantly smaller than one-CTV design group (FUSCC and SCH, *p* ≤ 0.006, *t* test). **(C)** Overview of CTV60s from one-CTV design and CTV54 from CTV54 from two-CTV design. CTV54 from SYSUCC also covers cervical area (white arrow in coronary figure). **(D)** The volume of CTV54 from FCH (two-CTV design) is significantly bigger than CTV60 from SCH (one-CTV design, *p* = 0.027), but if FUSCC is put into the analysis, the difference is not statistically significant (*p* = 0.131, *t* test).

Overall, the CTV60s in the one-CTV designs are larger than the CTV60s in the two-CTV design in all directions (Figure 1A). A quantitative volumetric comparison between the two groups is plotted in Figure 1B. Because CHCAM contoured CTV60 for the primary site and the high-risk cervical CTV (also 60 Gy) as one object (Figure 1A, right, white arrow), it was not included in this analysis. The results show that the volumes of the CTV60s in the two-CTV designs are systematically smaller than those of the CTV60s in the one-CTV designs, and such difference is statistically significant (*p* = 0.006, *t* test).

The two-CTV design has a low-risk CTV54 which might be volumetrically comparable to the CTV60 in the one-CTV design (Figure 1C). Therefore, we compared the volumes of the CTV60s in the one-CTV design to the CTV54s in the two-CTV design. CTV54s from SYSUCC were excluded from this analysis because their CTV54 and CTVln were contoured as a combined object (Figure 1C right, white arrow). The comparison was thus made between CTV54 of FCH and CTV60 of FUSCC and SCH. The results show that there is no significant difference among them (*p* = 0.131, *t* test). However, it appears that the CTV54 of FCH is relatively larger than all the five CTV60s of SCH, and the difference is statistically significant (*p* = 0.027, *t* test).

### Controversies on CTV Coverage

Both CTV60 of one-CTV design and CTV54 of two-CTV design are expected to cover the area harboring sub-clinical diseases. The variance in anatomical coverage of seven CTV60s from one-CTV design and seven CTV54s from two-CTV design show major controversies on the judgment of tumor spread possibility. Differences in the coverage of these 14 CTVs include the following:

1. There is great variance on coverage of the left half of pterygoid sinus and left cavernous sinus (Figure 2A).

**FIGURE 2:**
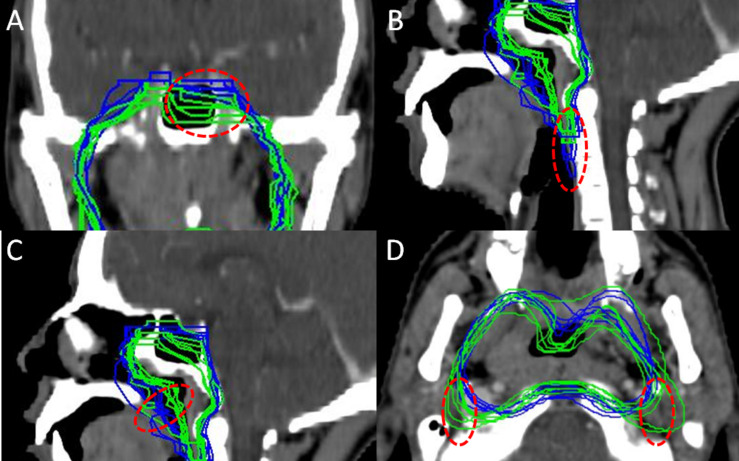
Some controversies among all contouring. **(A)** Coverage of pterygoid sinus and cavernous sinus at contra-lateral side. **(B)** Caudal border on posterior pharyngeal wall. **(C)** Coverage of air cavity. **(D)** Coverage of posterior space of styloid process.
2. A significant variance can be seen on the caudal border of CTV on the posterior pharyngeal wall. Some CTVs cover the posterior pharyngeal wall until midpoint of C3 (Figure 2B).
3. There is also remarkable difference in covering the air cavity of the nasopharynx (Figure 2C).
4. The failure at posterior space of styloid process was frequently seen in 2-D era due to brainstem avoidance. How much it should be covered for the prophylactic purpose in IMRT treatment still needs to be investigated (Figure 2D).

### CTV Coverage Displayed as Probability Map and Comparison to International CTV Consensus of NPC

**FIGURE 3:**
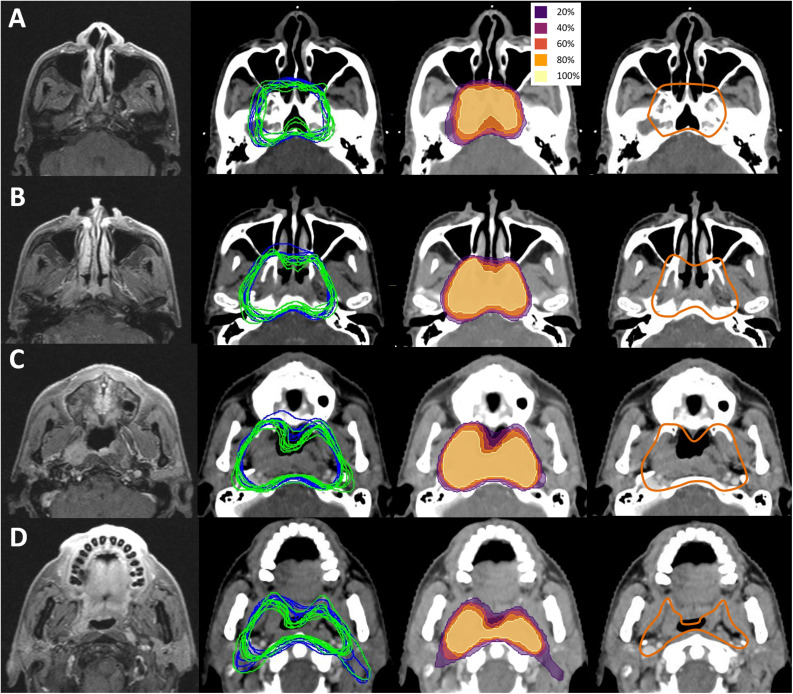
CTV coverage displayed as probability map and comparison to international CTV consensus of NPC. Left panel, T1 contrast enhanced MR images, middle left panel, original contouring of CTV60 of one-CTV design (blue lines) and CTV54 of two-CTV design (green lines), middle right panel, heat map showing involved probability of anatomical area, right panel, CTV recommended by the international consensus. Note the high consistence between contouring of Chinese physicians and recommendation of international consensus. However, some differences were also appreciable. **(A)** The difference in covering maxillary sinus at contra-lateral side. Note most Chinese physicians covered less than the consensus. **(B)** Difference in covering parapharyngeal space and lateral pterygoid plate at middle plane of maxillary sinus. Most Chinese physicians covered less than consensus at contra-lateral side. **(C)** Difference in covering parapharyngeal space and lateral pterygoid plate at hard plate level. Pterygoid process at contra-lateral side was spared, and medial edge of lateral pterygoid plate was used as left border by most physicians. **(D)** Difference in covering parapharyngeal space and posterior space of styloid process. At this level, the front part of the parapharyngeal space was spared for both sides by most Chinese physicians.

The probability of any given voxel being included for prophylactic purpose in all 14 contouring was calculated and displayed as a heat map that could immediately show the agreement and controversies visually. Figure 3 shows this map for four representative sections (the full map is available in Supplementary Material). For comparison, the CTV suggested by the international consensus were also delineated following a template provided by the consensus (right column) (3). According to the original contouring shown in the left column and the heat map in the middle left column, it can be seen that the coverage of prophylactic volume is largely consistent with the international consensus (right column). However, some minor differences were also noted. For the left side without tumor involvement, most Chinese physicians took the posterior wall of maxillary sinus as the front edge of CTV, whereas the consensus covers 5 mm of the posterior part of the maxillary sinus (Figures 3A,B). Coverage of parapharyngeal space of uninvolved side was also tailored by most Chinese physicians. Less pterygoid muscle was included in Chinese physicians’ contouring, whereas in the consensus, the lateral pterygoid plate and part of the pterygoid muscle are consistently covered, and full parapharyngeal space was covered even at the soft palate level, leading to a close margin to alveolar process (Figures 3B–D).

## Discussion

IMRT treatment for NPC was started in the late 1990s and early 2000s. The results published by the University of California San Francisco (UCSF) group in the early 2000s (8) have been acknowledged as the earliest reports of IMRT on NPC. The CTV proposed by UCSF (CTV59.4) covers the entire nasopharyngeal mucosa, posterior third of maxillary sinus and nasal cavity, clivus, parapharyngeal space, sphenoid sinus, and pterygoid fossae. This CTV design was also adopted by the subsequent RTOG 0615 trial with limited modifications on anterior and posterior border (9). The CTV definition used in early studies published by groups from endemic area of NPC (10, 11) were very similar to the one proposed by UCSF and RTOG 0615, all of which were inherited from field design of conventional radiotherapy.

Based on improvements in MR imaging and accumulating experience on IMRT, reduced volumes for CTVs and differential dose prescriptions within the CTV have been proposed (12–15). Lin et al. defined the GTV plus 0.5–1 cm margin as high-risk CTV (CTV60), with a 60 Gy prescription (16). Their low-risk CTV (CTV54, 54 Gy) covered the area that UCSF CTV59.4 covered but with reductions in almost all directions. The CTV volume in Lin’s study (CTV54) was 160.2 cc (range, 86.5–337.1), which was significantly smaller than the one in Sultanem’s study from UCSF (average 212 cc, range, 104–339). Groups from non-endemic areas also reported their results with reduced CTV volumes (17–19).

In China, an experts’ consensus on IMRT field design for NPC treatment has been established in 2010 (4). The CTV design was similar to the one proposed by Lin (16). In 2017, an international consensus for CTV delineation of NPC was published with significant volume reduction compared to RTOG 0615 (3). In this study, the prophylactic volume is very consistent among all physicians and is in agreement with the Chinese and the international consensus. One of the noticeable alterations that most physicians made was further shrinking the border at contra-lateral site. Similar adaptation was also reported by Sanford et al. (18), suggesting that reduction of treatment volume at un-involved site might be safe and without loss in tumor control.

Currently, for most centers in China, CTV is not treated with full dose, and indeed in this study only one center adopted this approach, with a prescribed dose to the CTV of 66 Gy. There are two approaches to CTV definition in China: the one-CTV design is consistent with the recommendations of the international consensus, whereas the two-CTV design substantially follows the principles of the 2010 Chinese consensus. In this study, we showed that there is no fundamental difference in terms of prophylactic volumes between these two strategies. The two-CTV design has the advantage of reducing normal tissue toxicity because of its relative smaller 60 Gy coverage. However, it should be noted that the centers deploying two-CTV designs are all in south China, where the NPC endemic area is located. In these areas, over 90% of NPCs are Epstein–Barr virus (EBV)-associated, undifferentiated, non-keratinized carcinomas, whereas in non-endemic areas, only about 30% of NPCs are differentiated non-keratinized carcinomas (20), and a significant fraction of them are not EBV-associated (21). Dose deintensification on these tumors should be performed with caution because they may have a considerably different response to treatment compared to tumors in endemic areas. The different distribution of physicians involved in the different centers is a limitation of this study, but it should be noted that the order of magnitude of intra-institution variations are on average comparable to inter-institution variations (see Figure 1), which means that although internal guidelines improve consistency, still the controversies discussed in this work apply.

Our work also displayed controversies on CTV coverage among Chinese physicians. Currently, there is no clear consensus on these questions, and further clinical studies should be undertaken to clarify them. Some controversies, however, seem to be caused by personal preference of physicians or by institutional conventions. For instance, for the caudal border, some contours were as low as C3 level. It has already been proven that the central group of retropharyngeal nodes is rarely involved between C2 and hyoid bone (22). It seems unnecessary to cover so much posterior wall of oropharynx for a tumor located within the nasopharynx. We generated a heat map of CTV coverage based on all 14 contours. For any controversies, an over-60% agreement for coverage of any given site should be considered as an acceptable choice (Supplementary Material).

This is the first study that directly compares contouring strategies among different physicians from different centers in China. We showed that the coverage of prophylactic area was in high agreement among all centers that participated. However, in centers from endemic areas, reduced dose to CTV has been routinely applied. The study also found disagreements on the coverage of multiple sites. Some of them need to be investigated by clinical studies. However, some variations could be minimized when unmotivated personal preferences are removed. Recently, automated contouring of NPC GTV using machine learning yielded promising results (23). Artificial intelligence (AI)-based innovative tools are now expected to help reduce inter-observer and inter-institution variance on CTV delineation in the near future.

Standardization of methods is fundamental to acquire a reliable guidance that can be adapted to each specific case. Otherwise, variability in treatments and in data acquisition produces non-homogeneous results which ultimately will affect the soundness of the research work.

## Data Availability Statement

The raw data supporting the conclusions of this article will be made available by the authors, without undue reservation.

## Ethics Statement

The studies involving human participants were reviewed and approved by Ethical Committee of the Sichuan Cancer Hospital. The patients/participants provided their written informed consent to participate in this study.

## Author Contributions

SZ, SY, PX, YX, GZ, XO, RW, ML, XW, SL, J-LY, YS, and CH collected data from participating centers. DF and JD performed the image processing tasks and produced the probability maps. SZ and JL designed the study and wrote the draft of the manuscript. All authors reviewed and corrected the draft.

## Conflict of Interest

The authors declare that the research was conducted in the absence of any commercial or financial relationships that could be construed as a potential conflict of interest. The handling editor declared a past co-authorship with one of the authors DF.

## Footnotes

1 http://insight-journal.org/browse/publication/887
