# Supplementary material for: Variations of Clinical Target Volume Delineation for Primary Site of Nasopharyngeal Cancer Among Five Centers in China
Source: Front Oncol. 2020 Aug 20;10:1572. doi: 10.3389/fonc.2020.01572 (PMC7468394; doi:10.3389/fonc.2020.01572)
Supplement: Supplementary file 1 [file Presentation_1.pdf]

CTVs contoured by 14 physicians in China for primary site of a T3N2M0 NPC

presented as persentile agreement

Centers participated
National Cancer Center of China
Fudan University Shanghai Cancer Center
Sichuan Cancer Hospital
Sun Yat-Sen University Cancer Center
Fujian Cancer Hospital

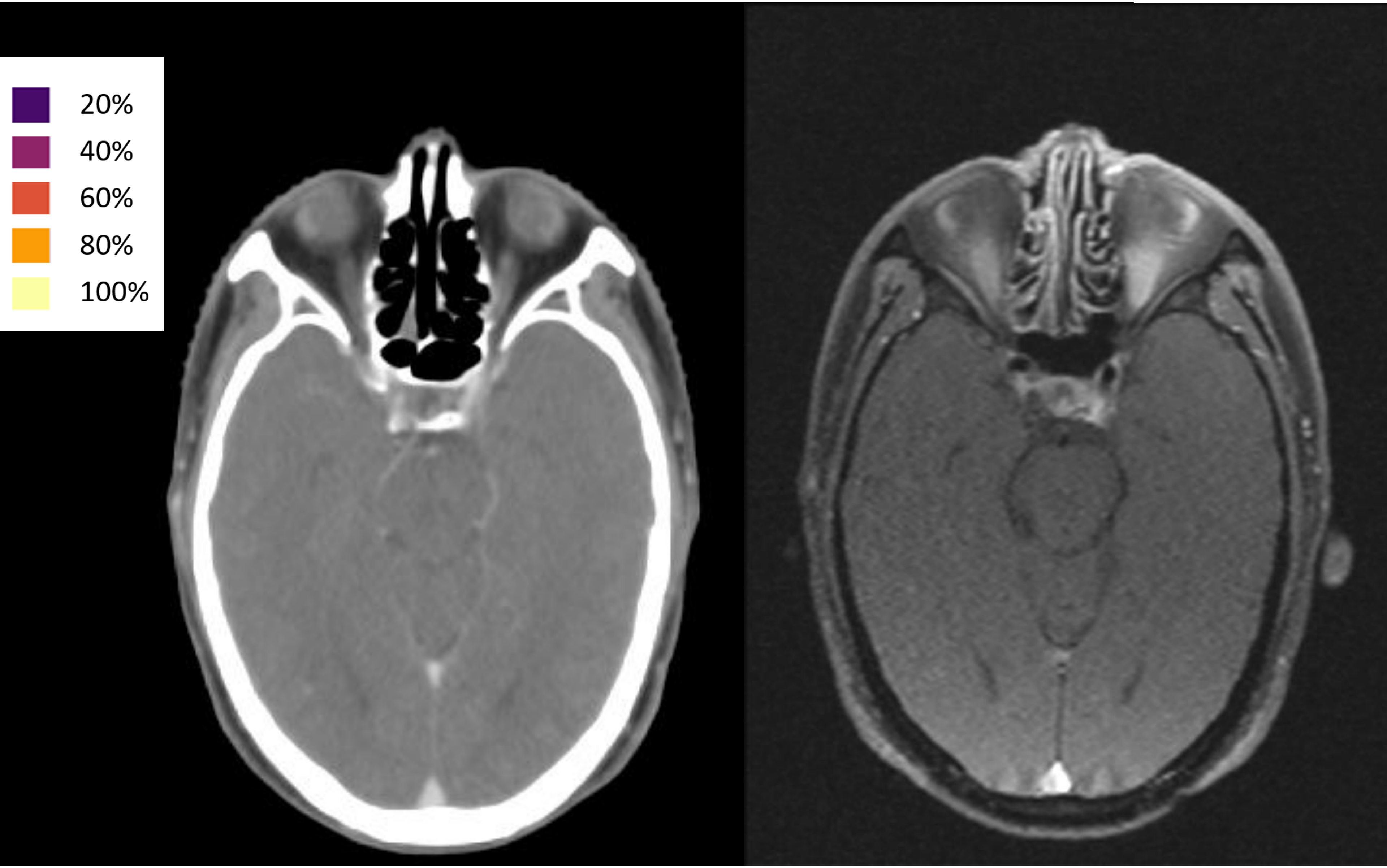
20%
40%
60%
80%
100%

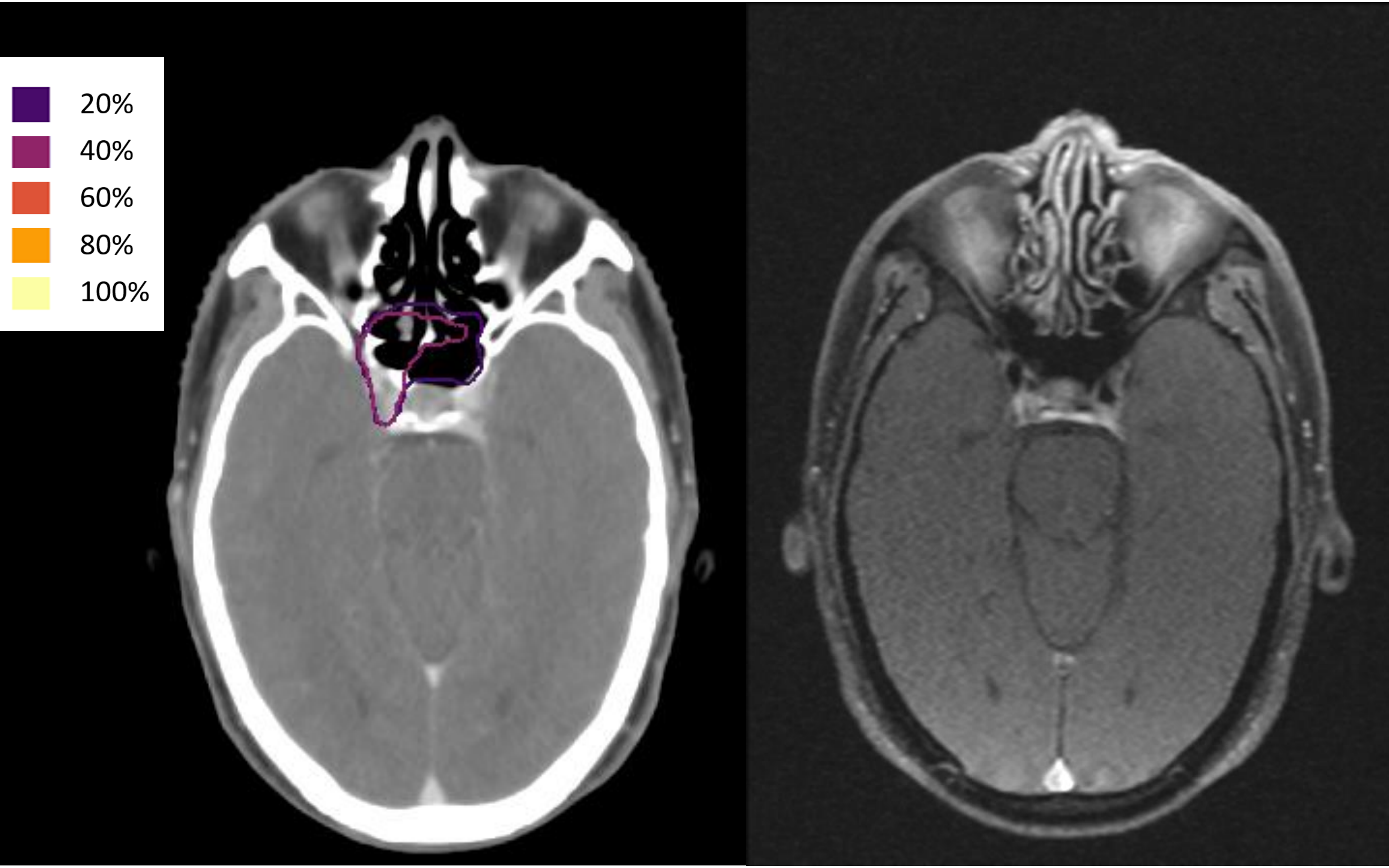
20%
40%
60%
80%
100%

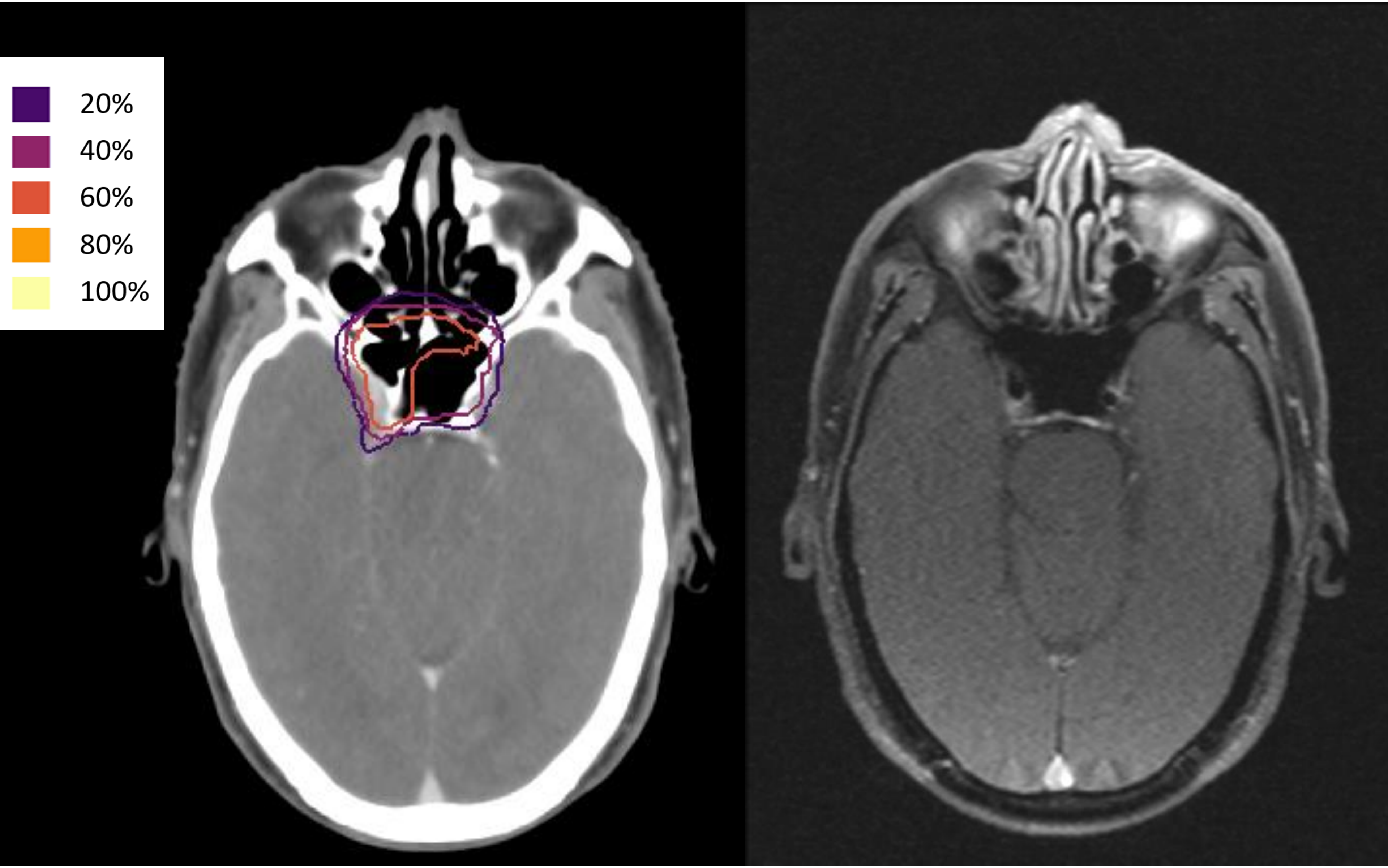
20%
40%
60%
80%
100%

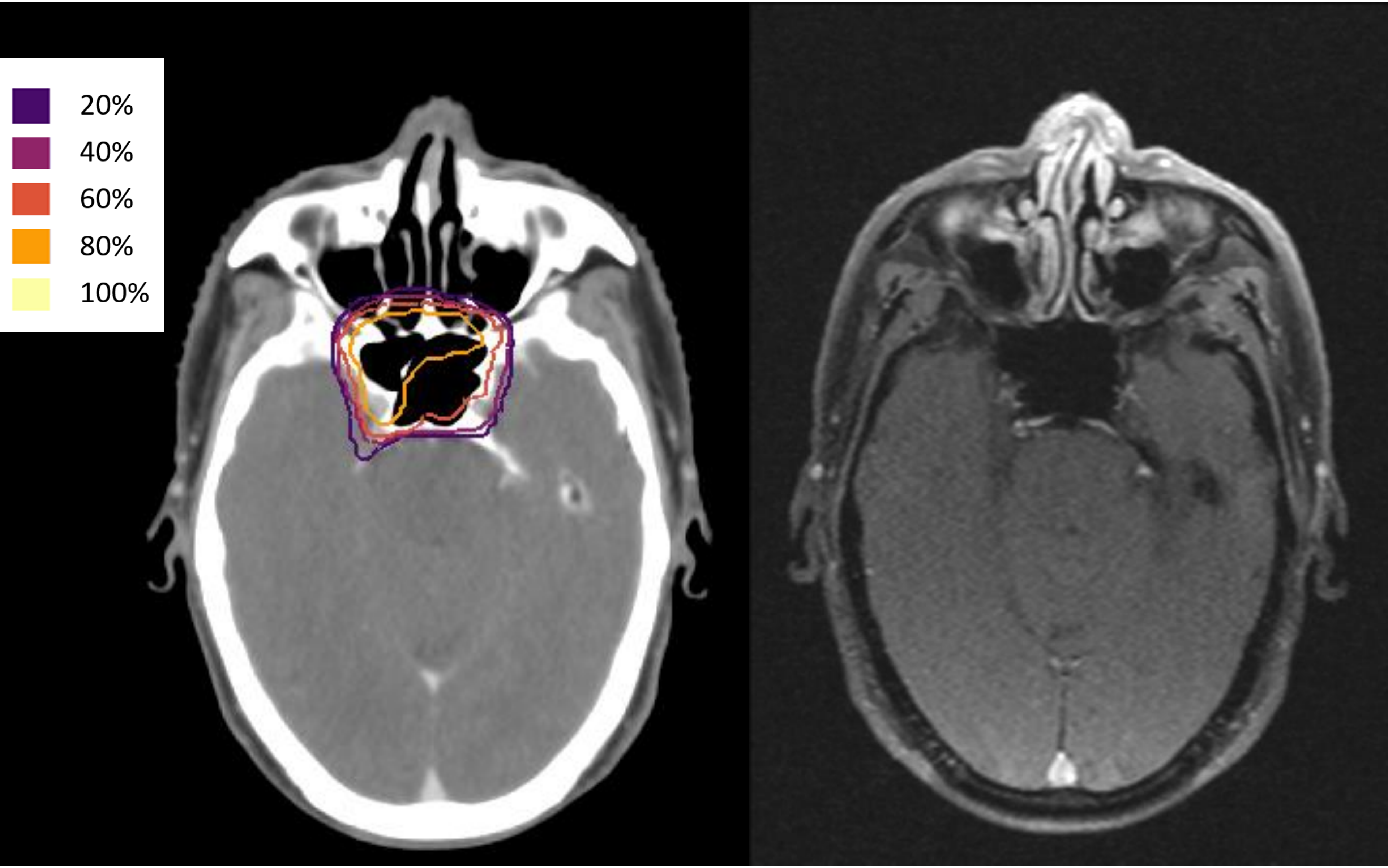
20%
40%
60%
80%
100%

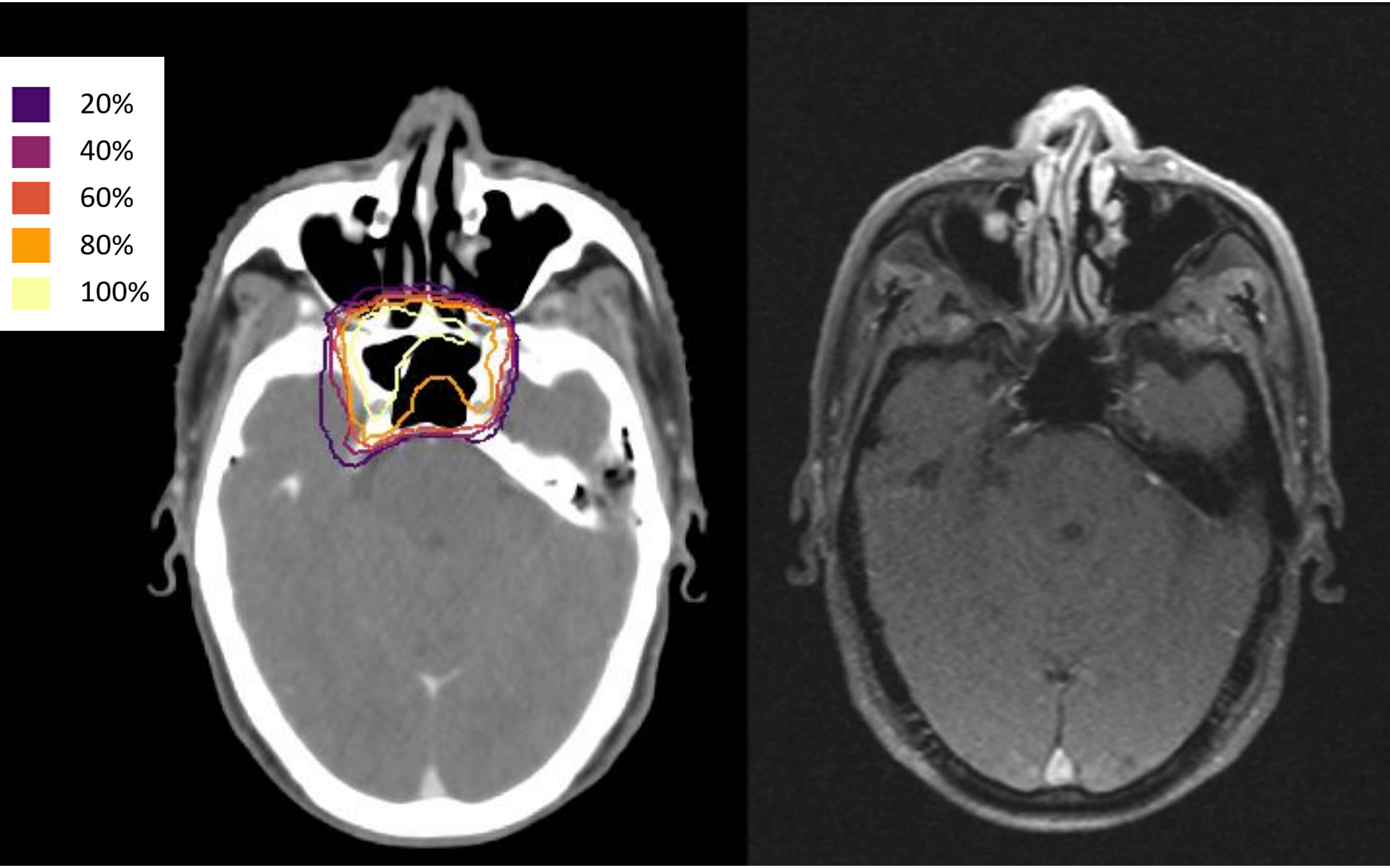
20%
40%
60%
80%
100%

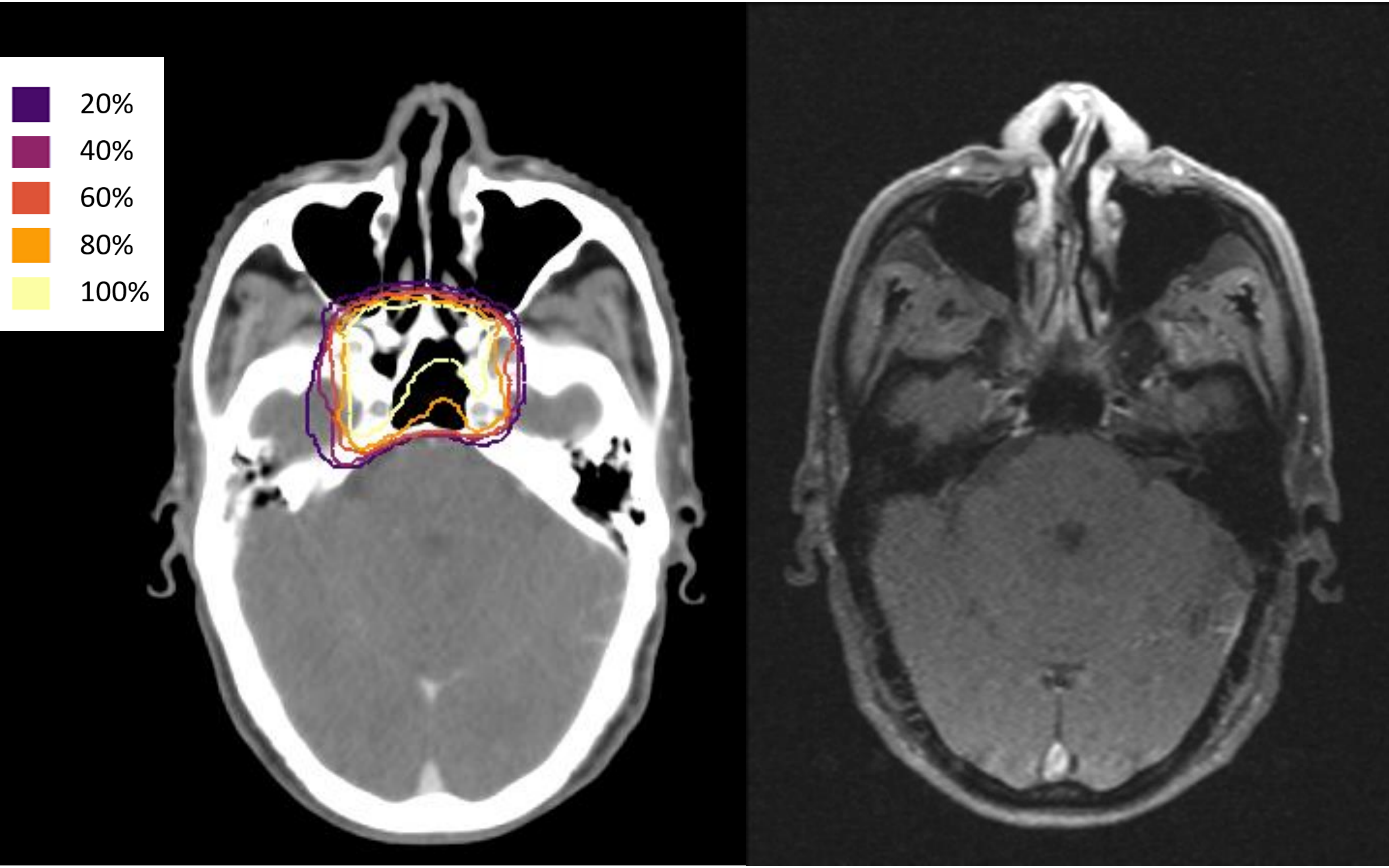
20%
40%
60%
80%
100%

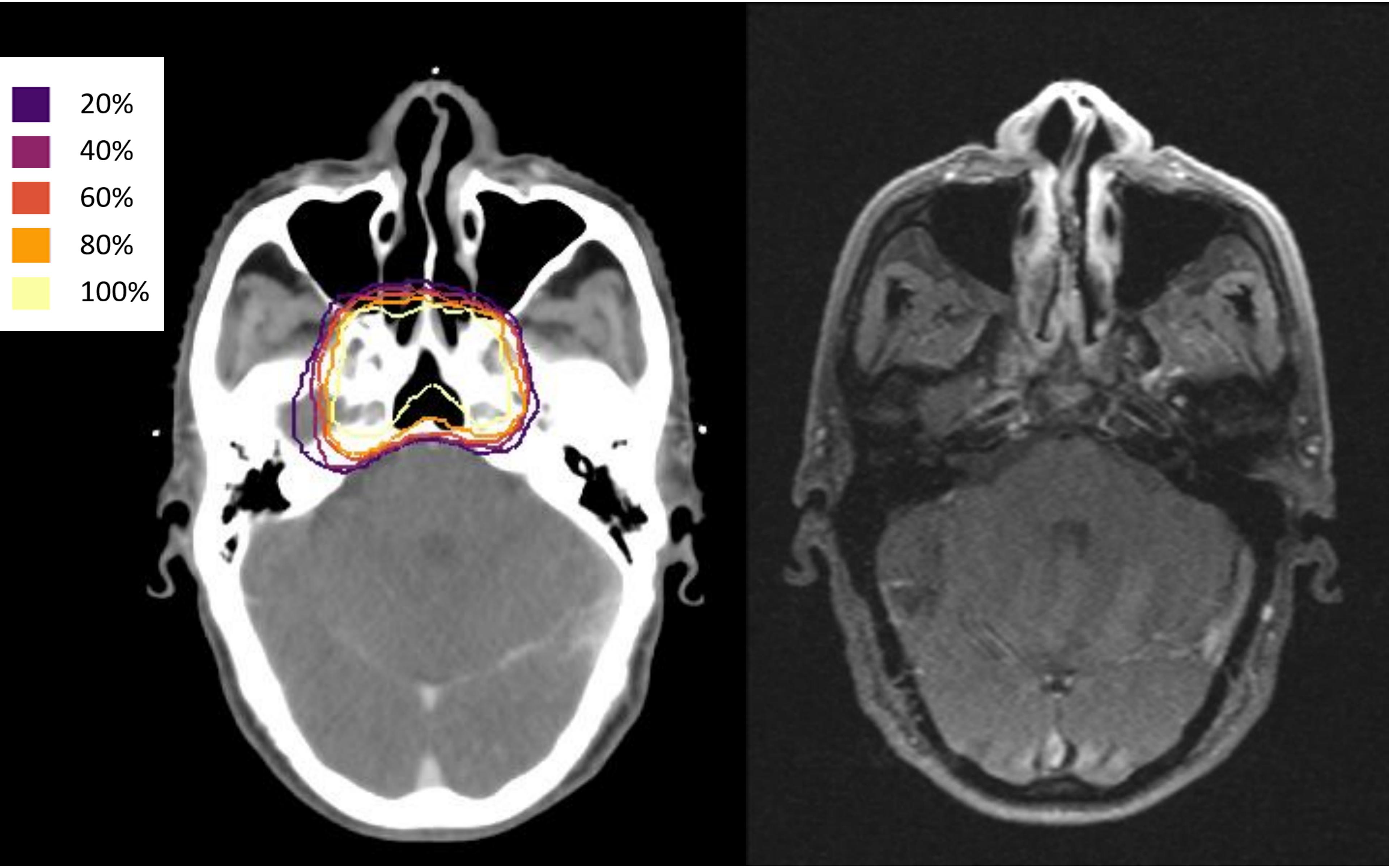
20%
40%
60%
80%
100%

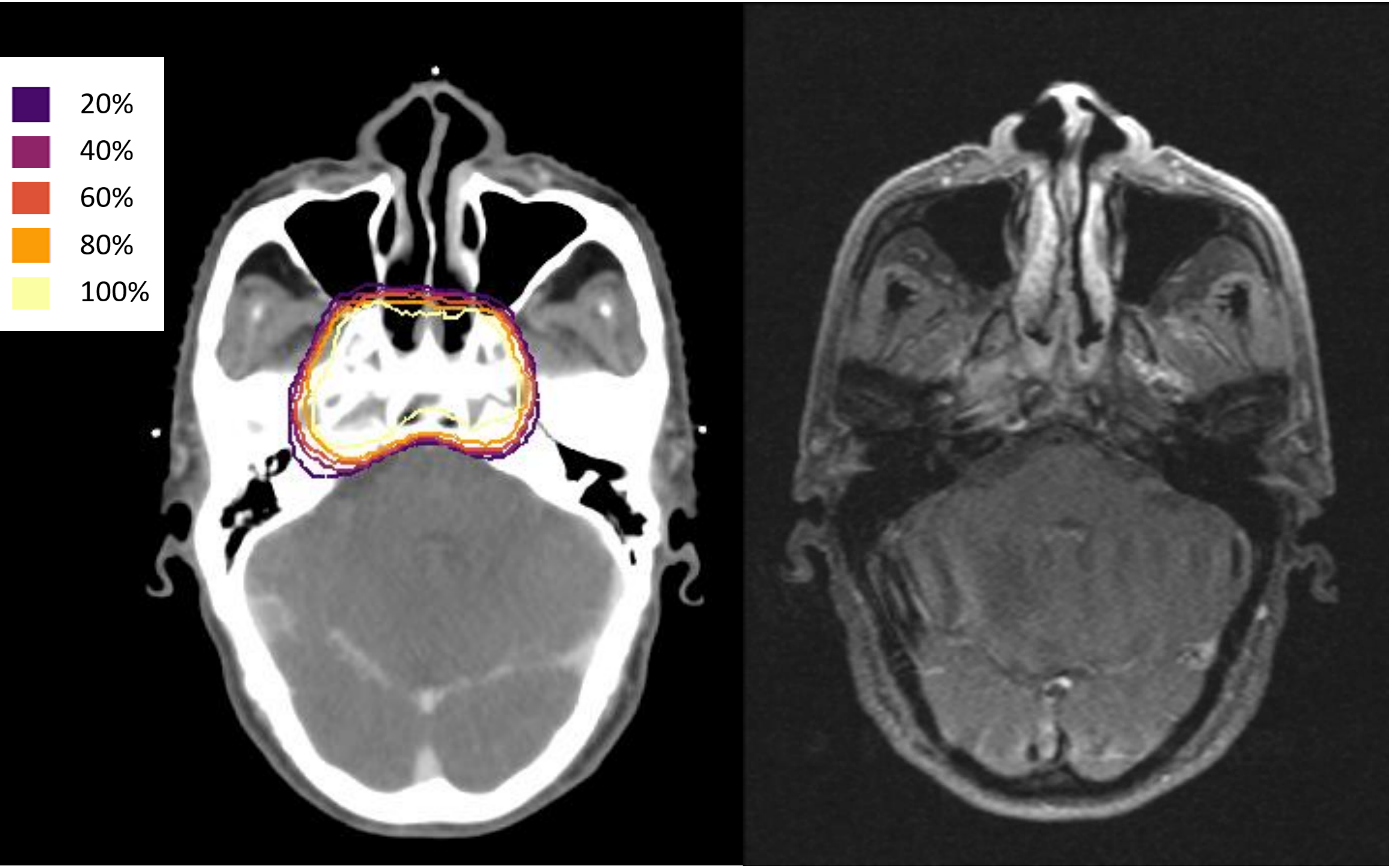
20%
40%
60%
80%
100%

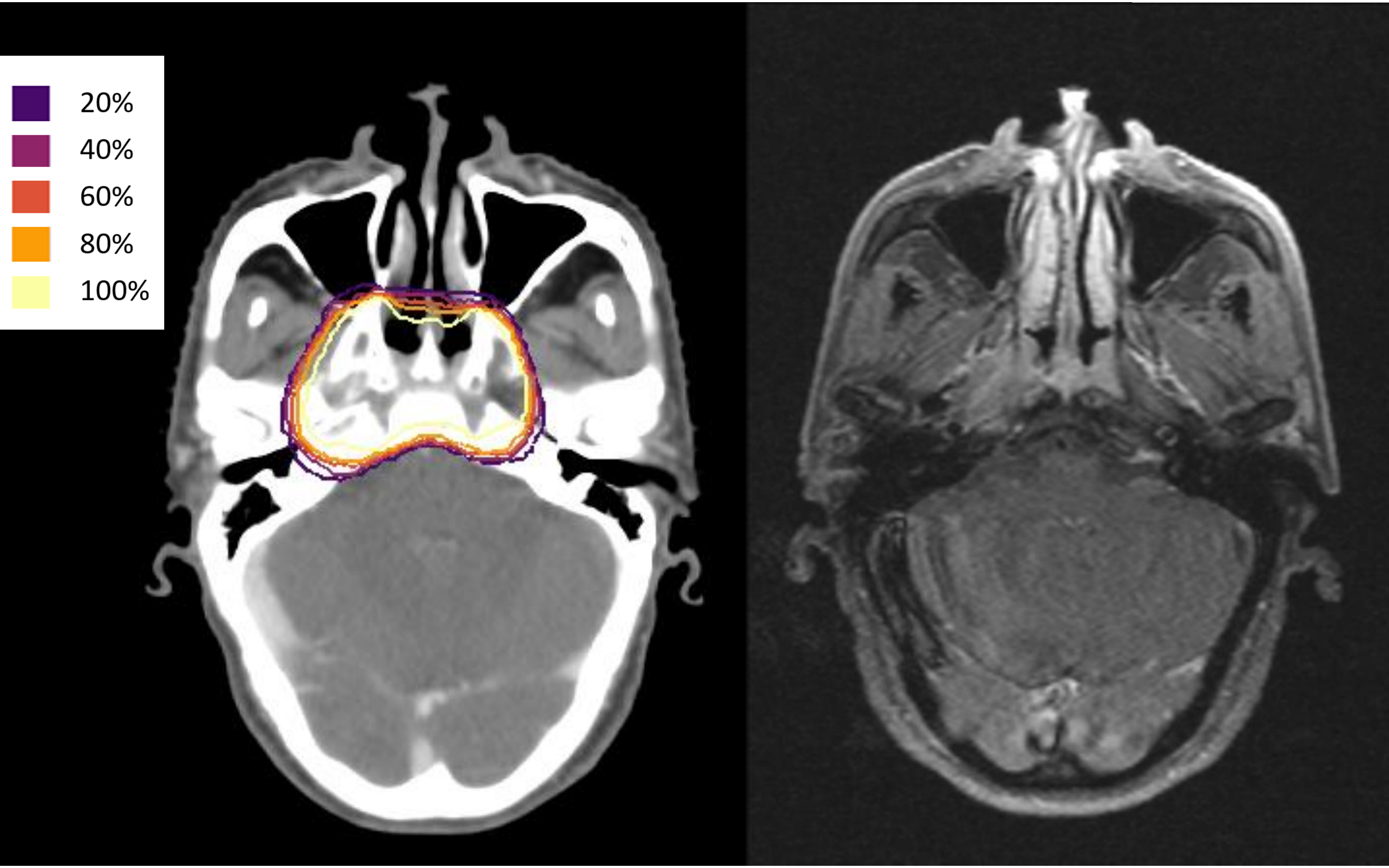
20%
40%
60%
80%
100%

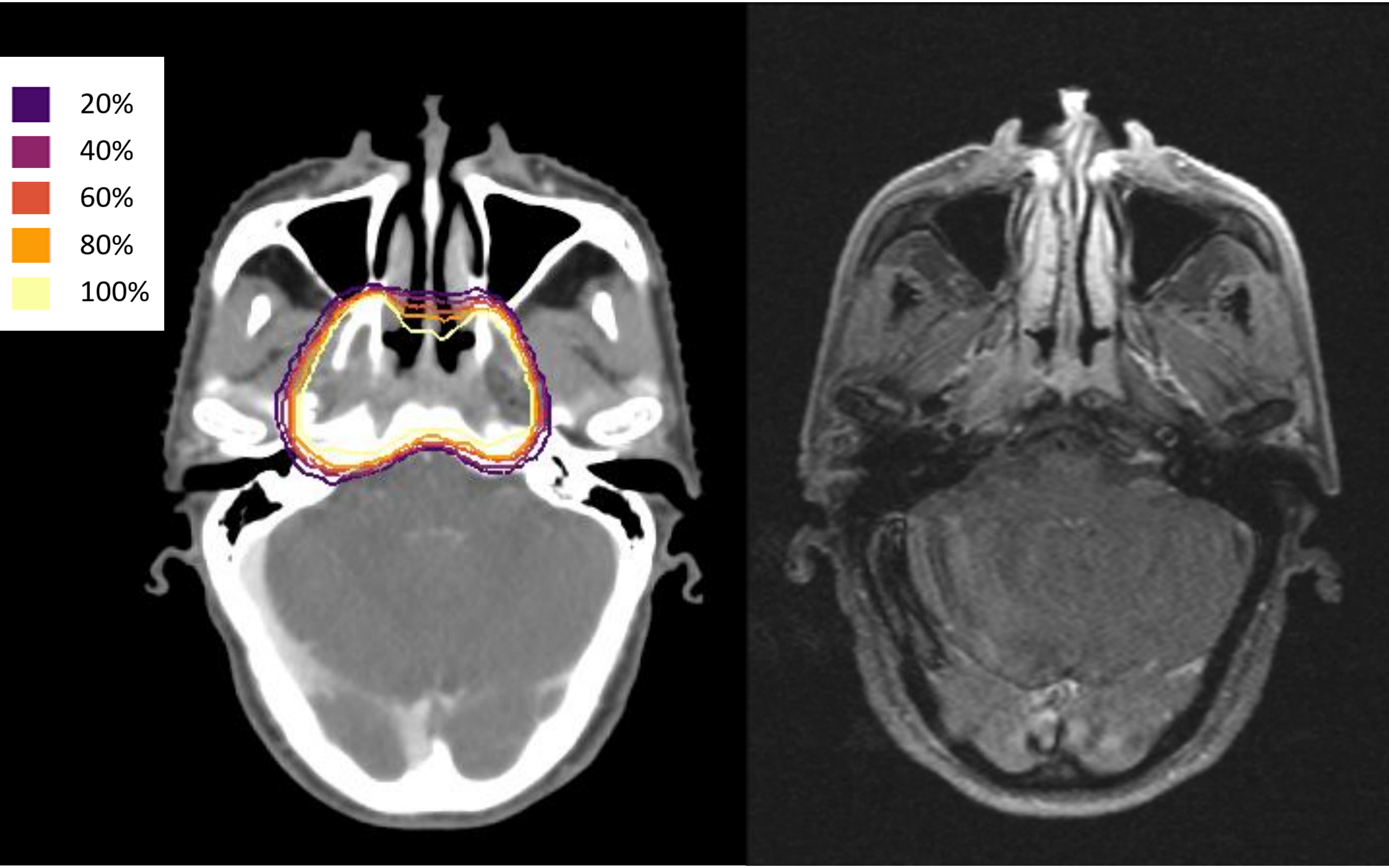
20%
40%
60%
80%
100%

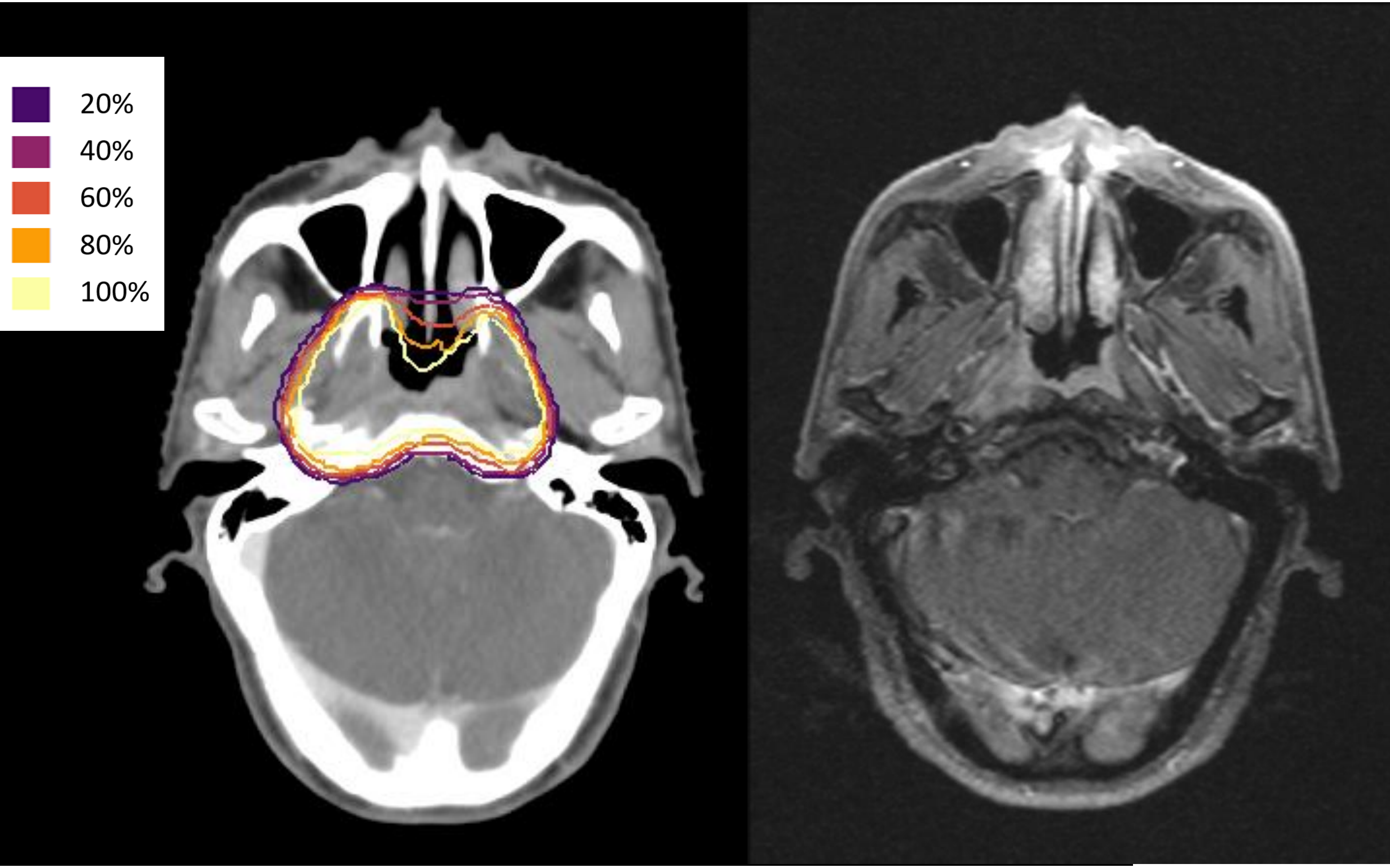
20%
40%
60%
80%
100%

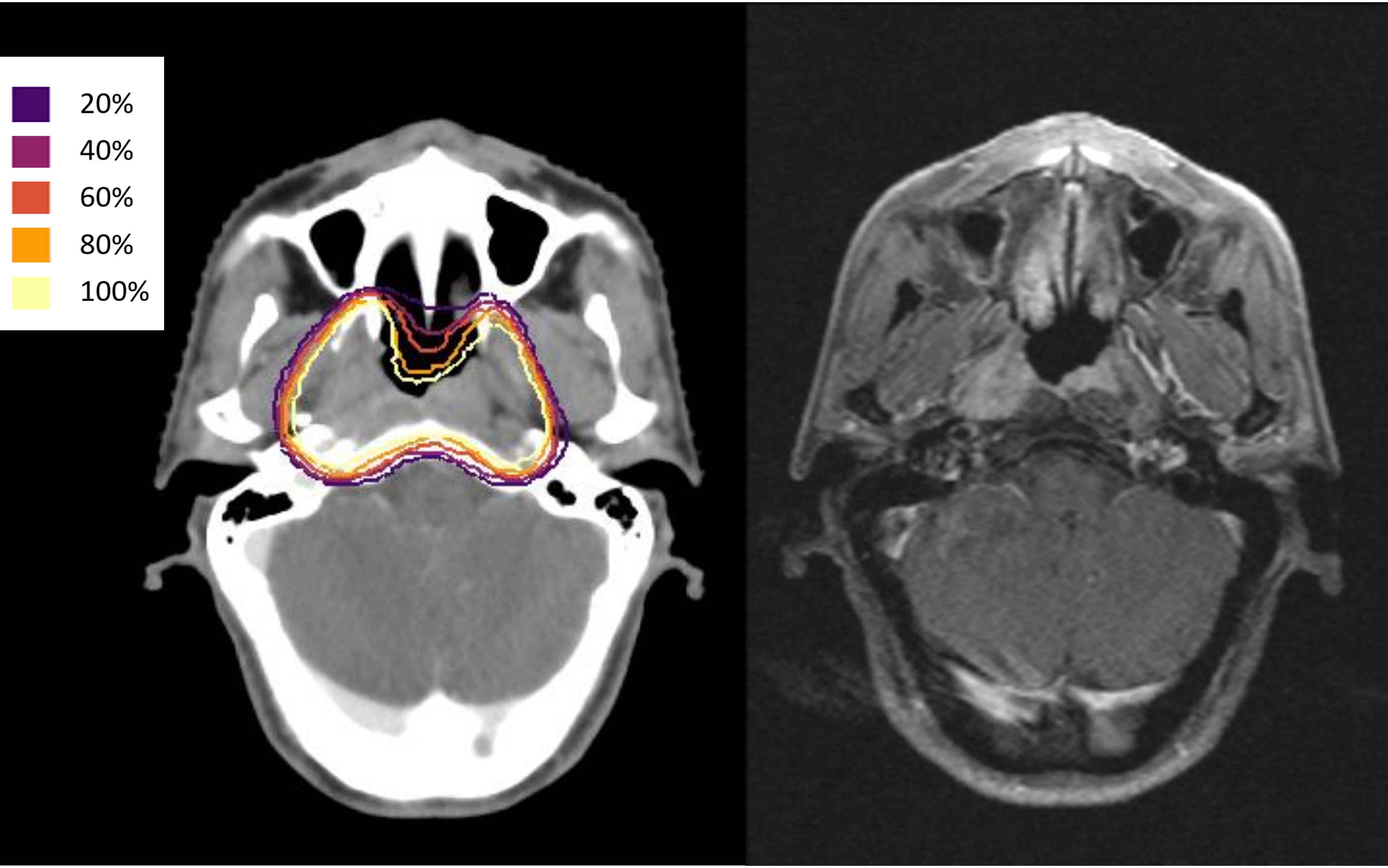
20%
40%
60%
80%
100%

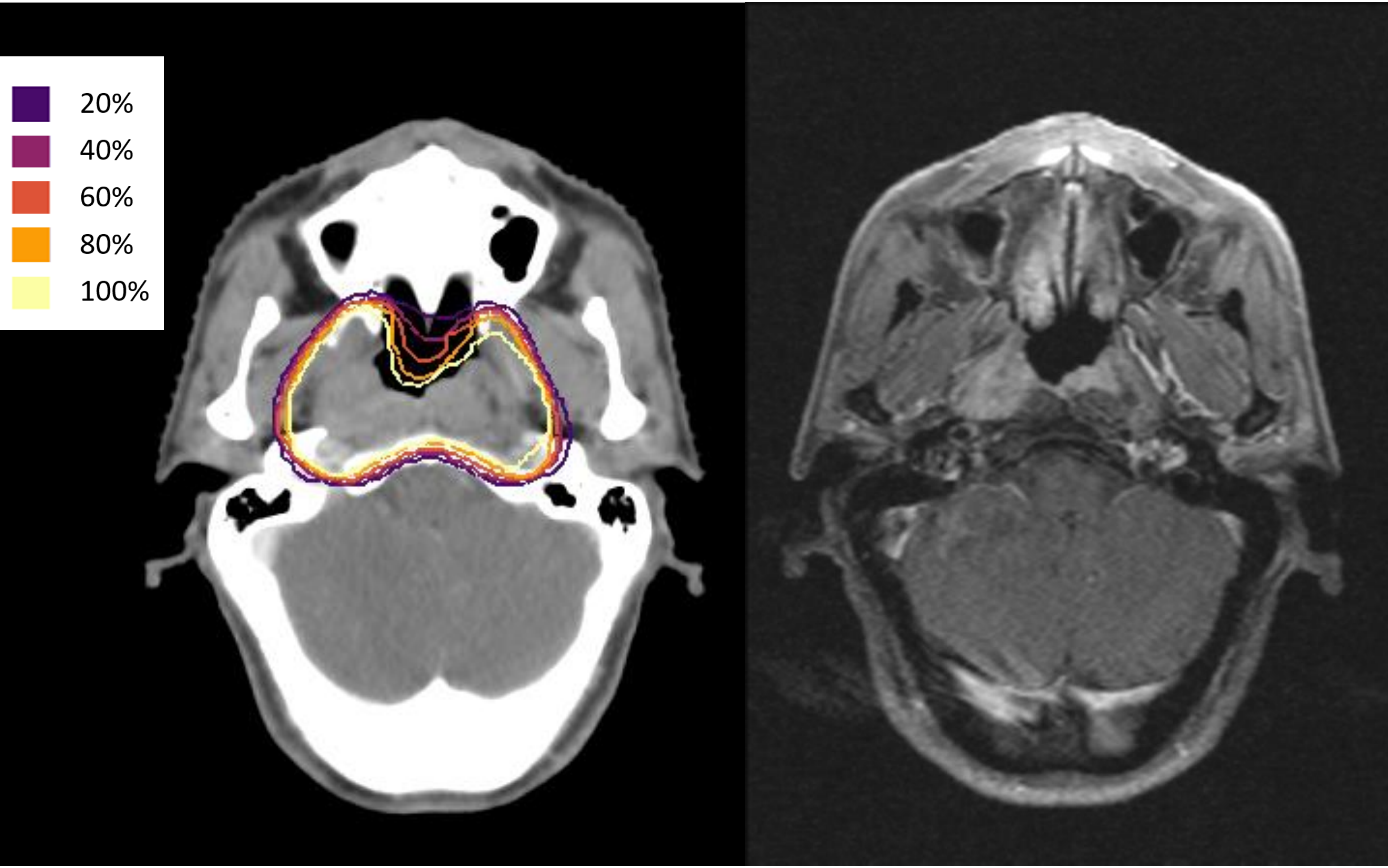
20%
40%
60%
80%
100%

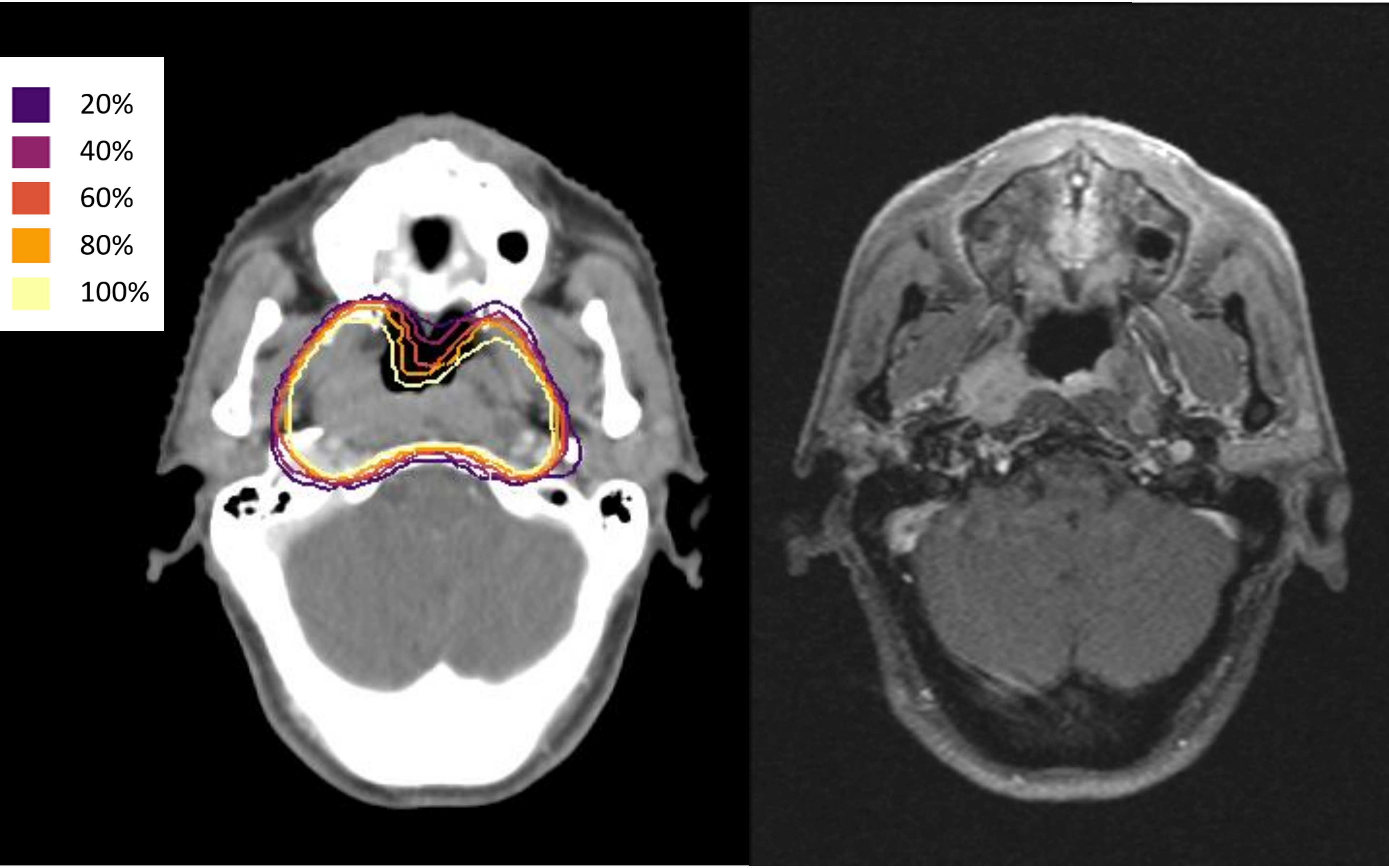
20%
40%
60%
80%
100%

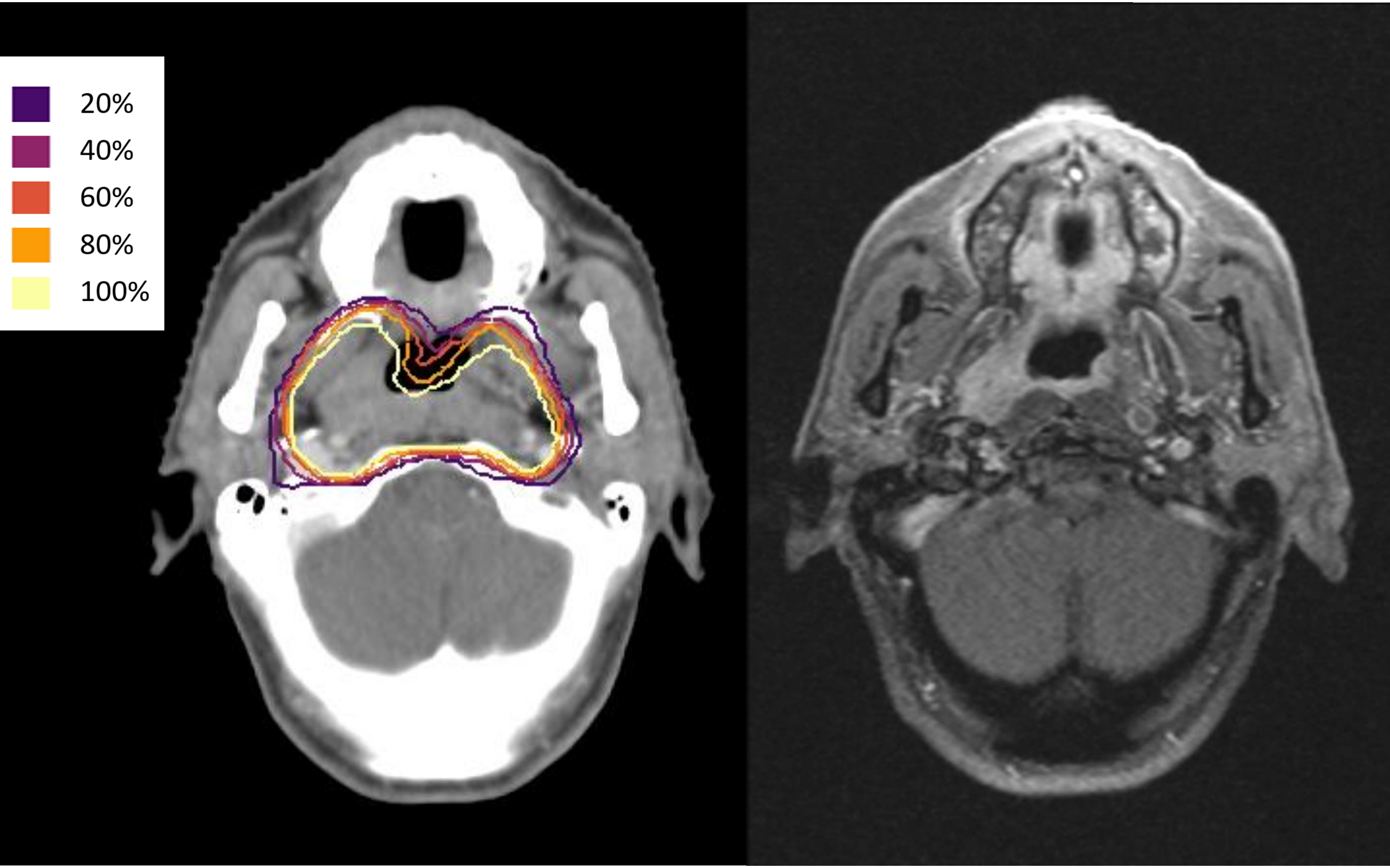

20%
40%
60%
80%
100%

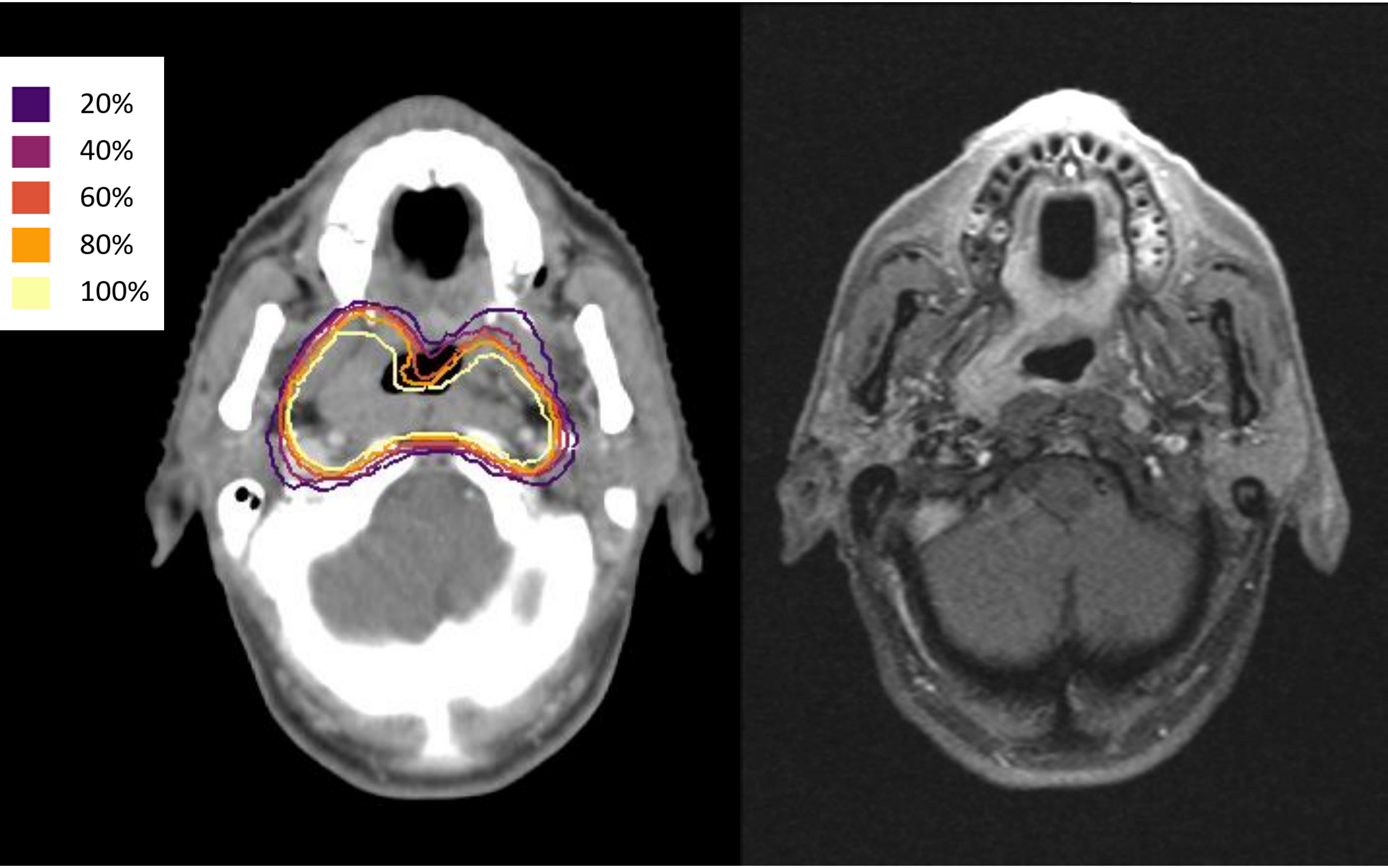

20%
40%
60%
80%
100%

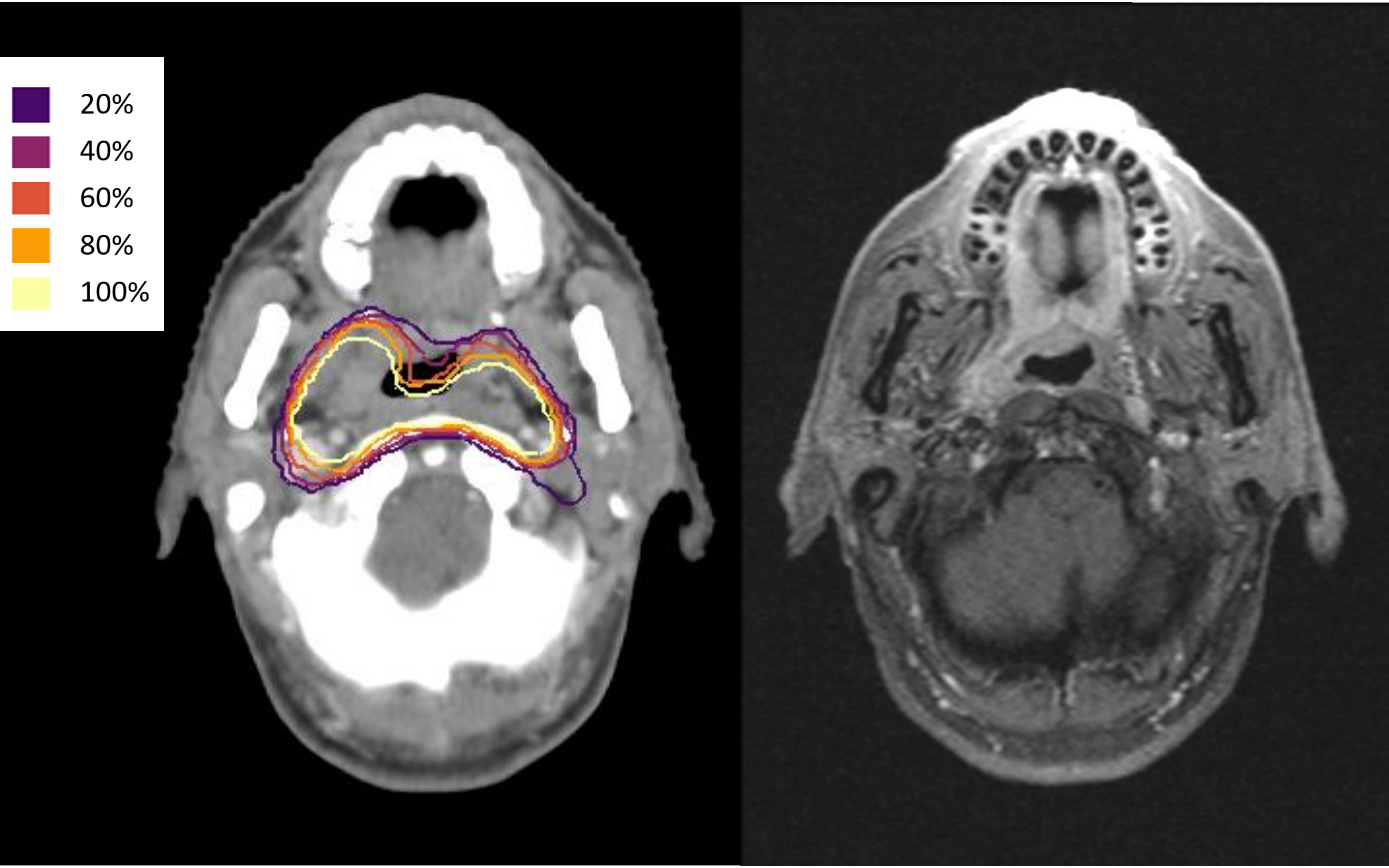
20%
40%
60%
80%
100%

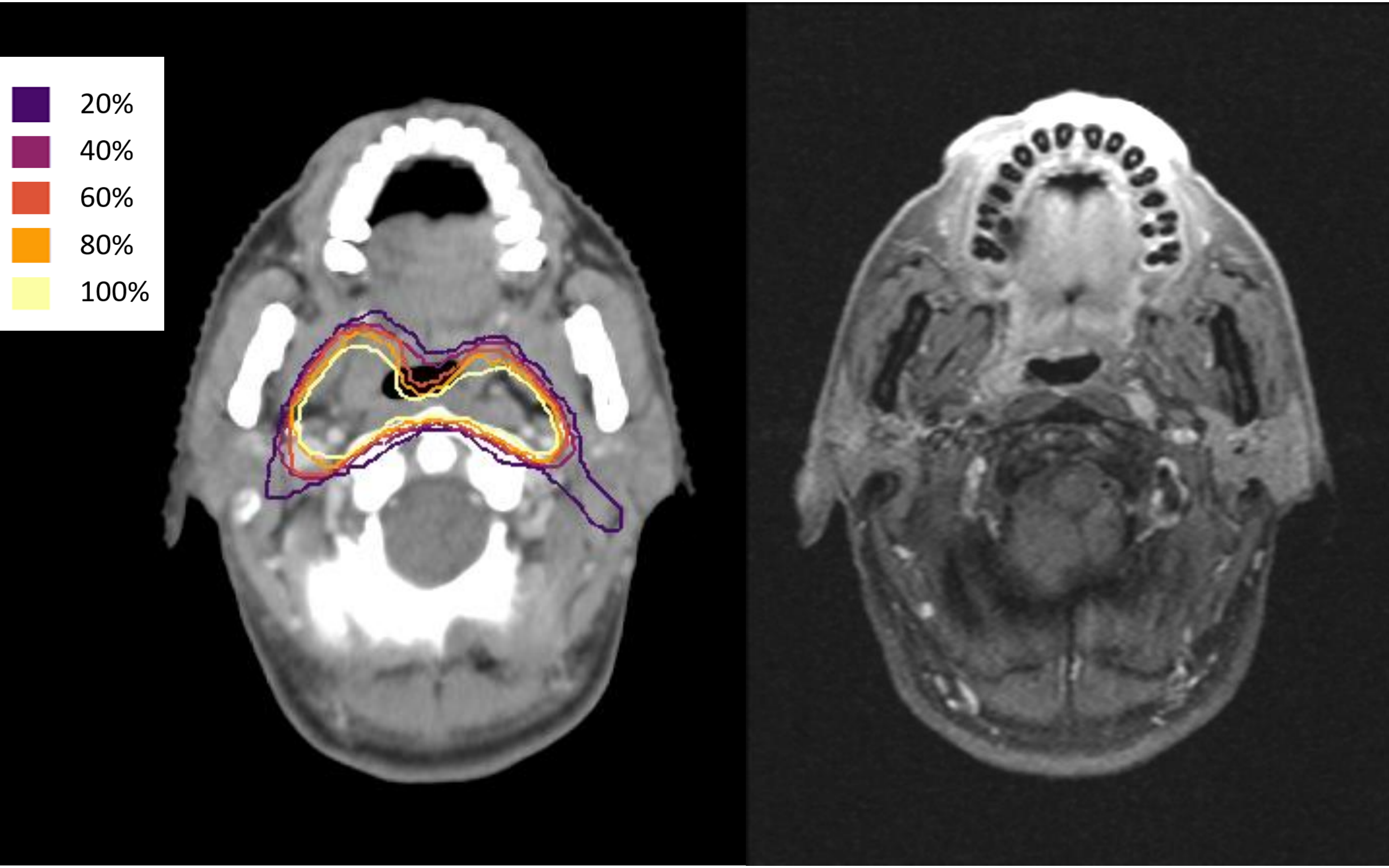

20%
40%
60%
80%
100%

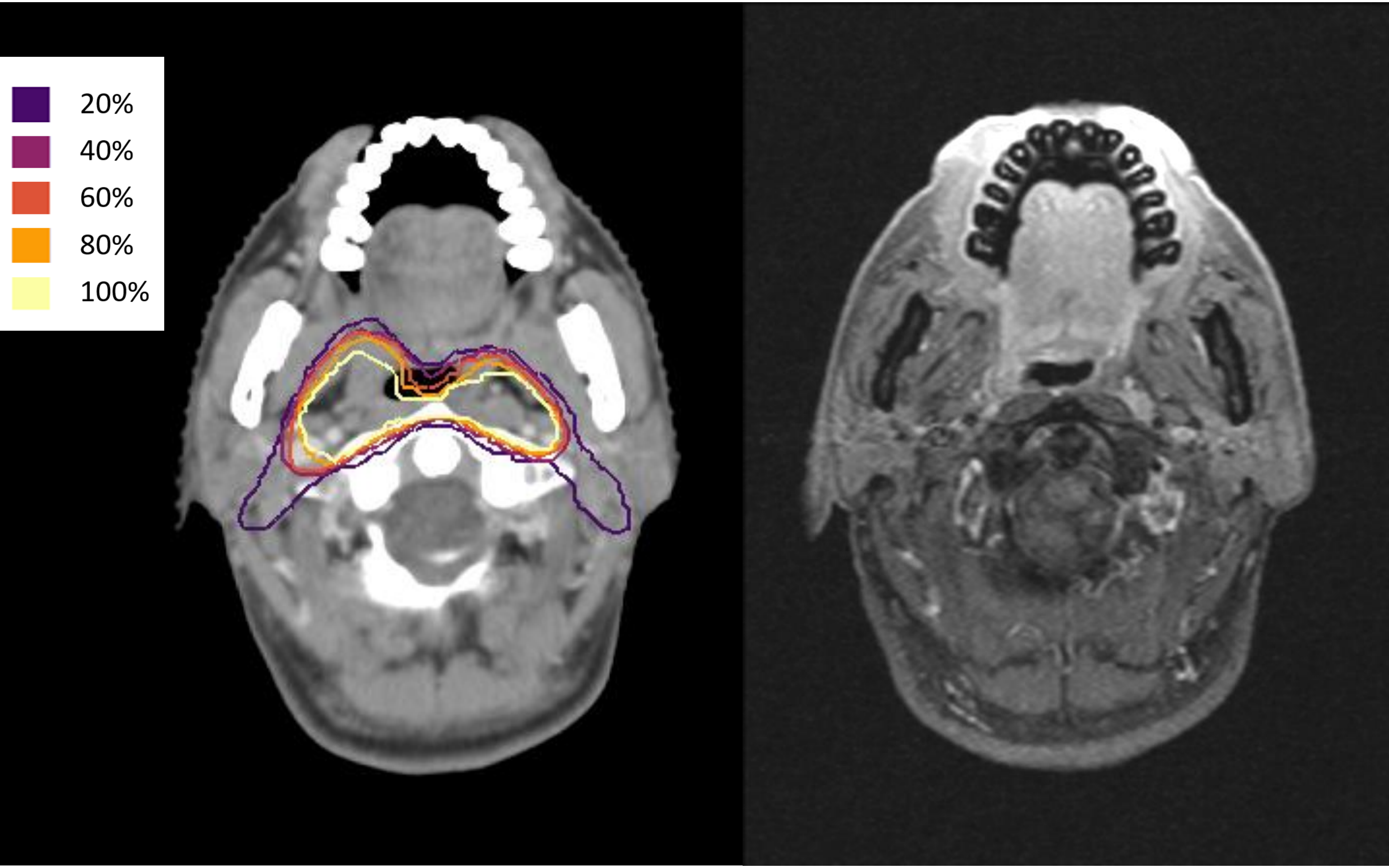

20%
40%
60%
80%
100%

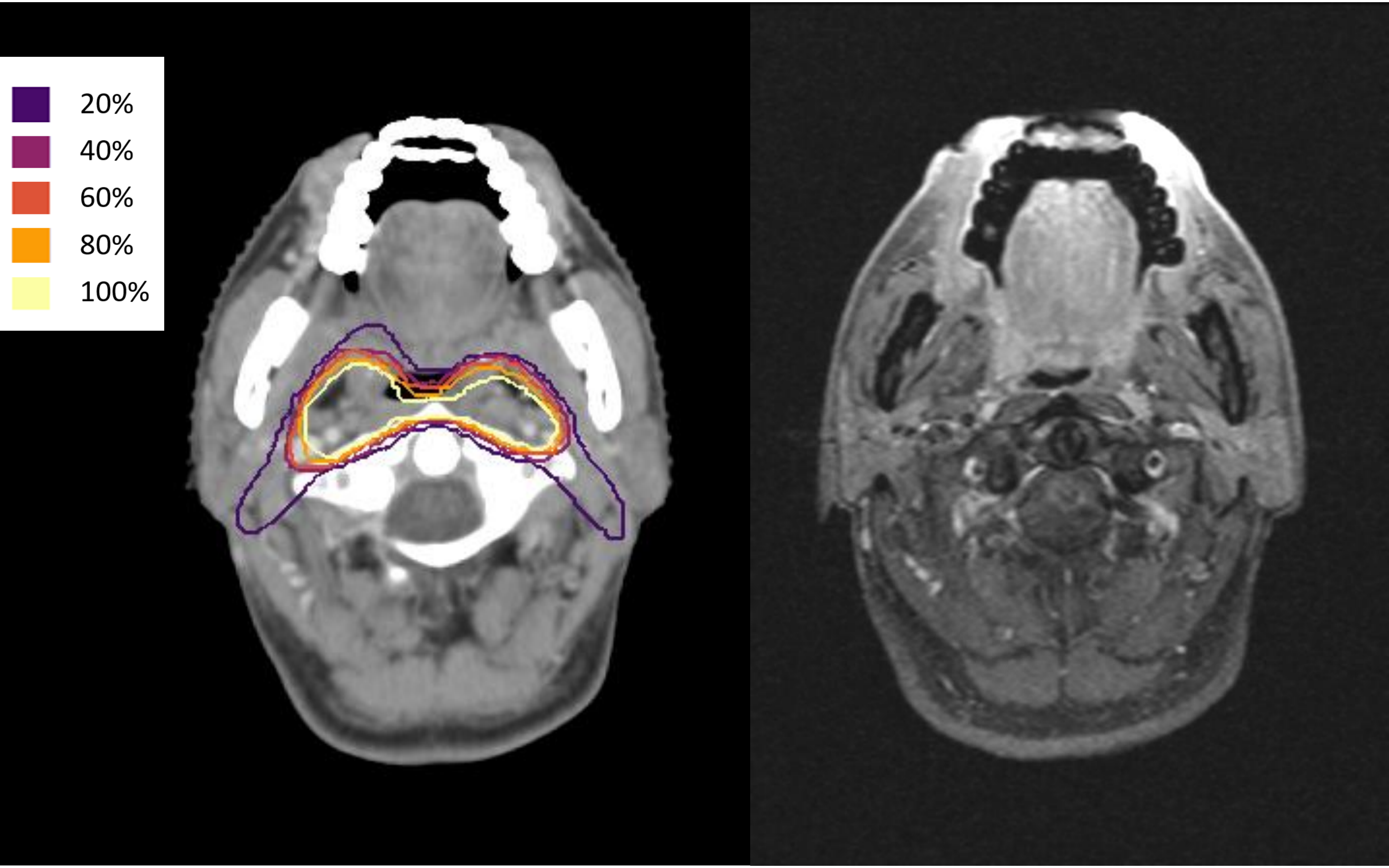
20%
40%
60%
80%
100%

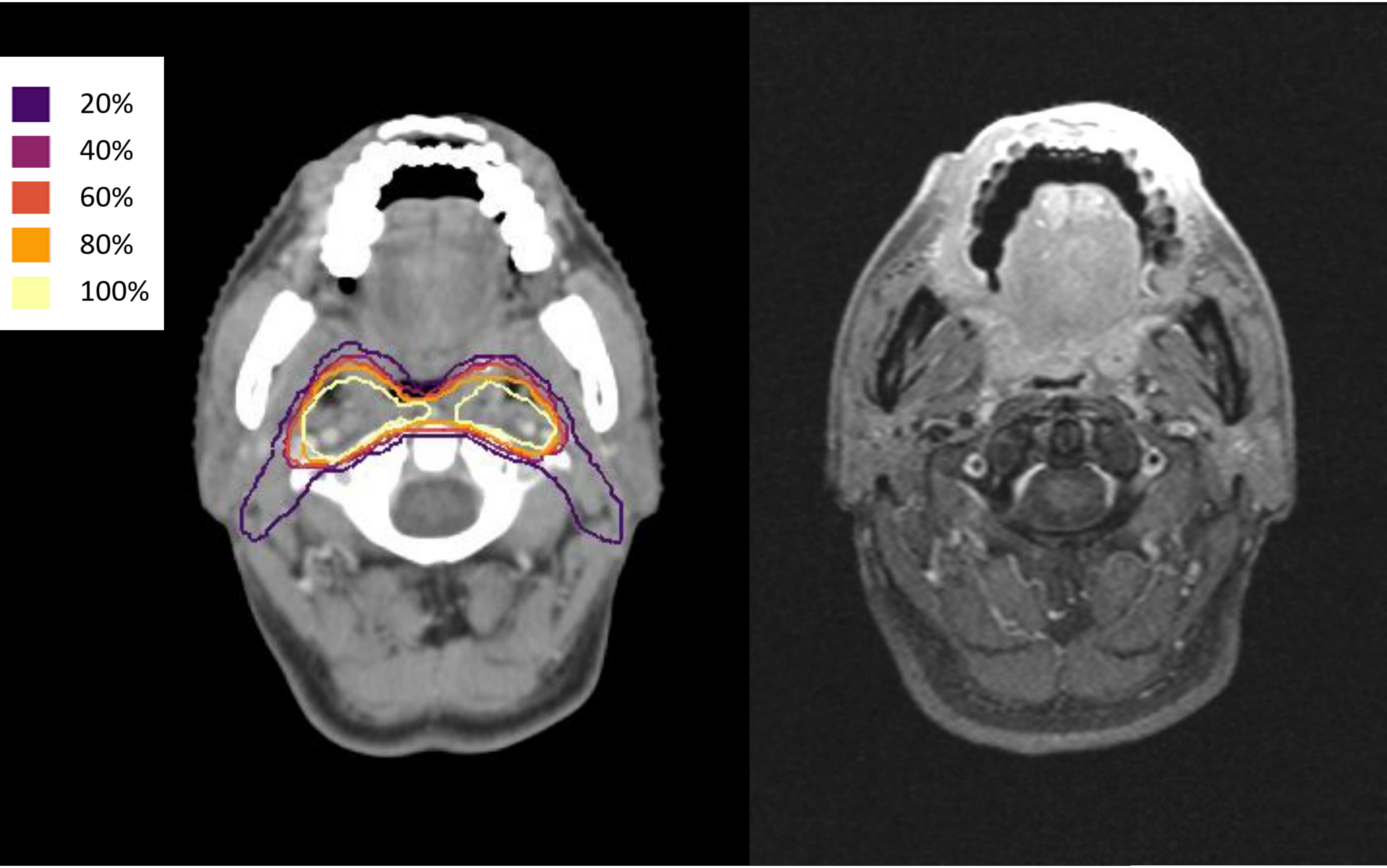
20%
40%
60%
80%
100%

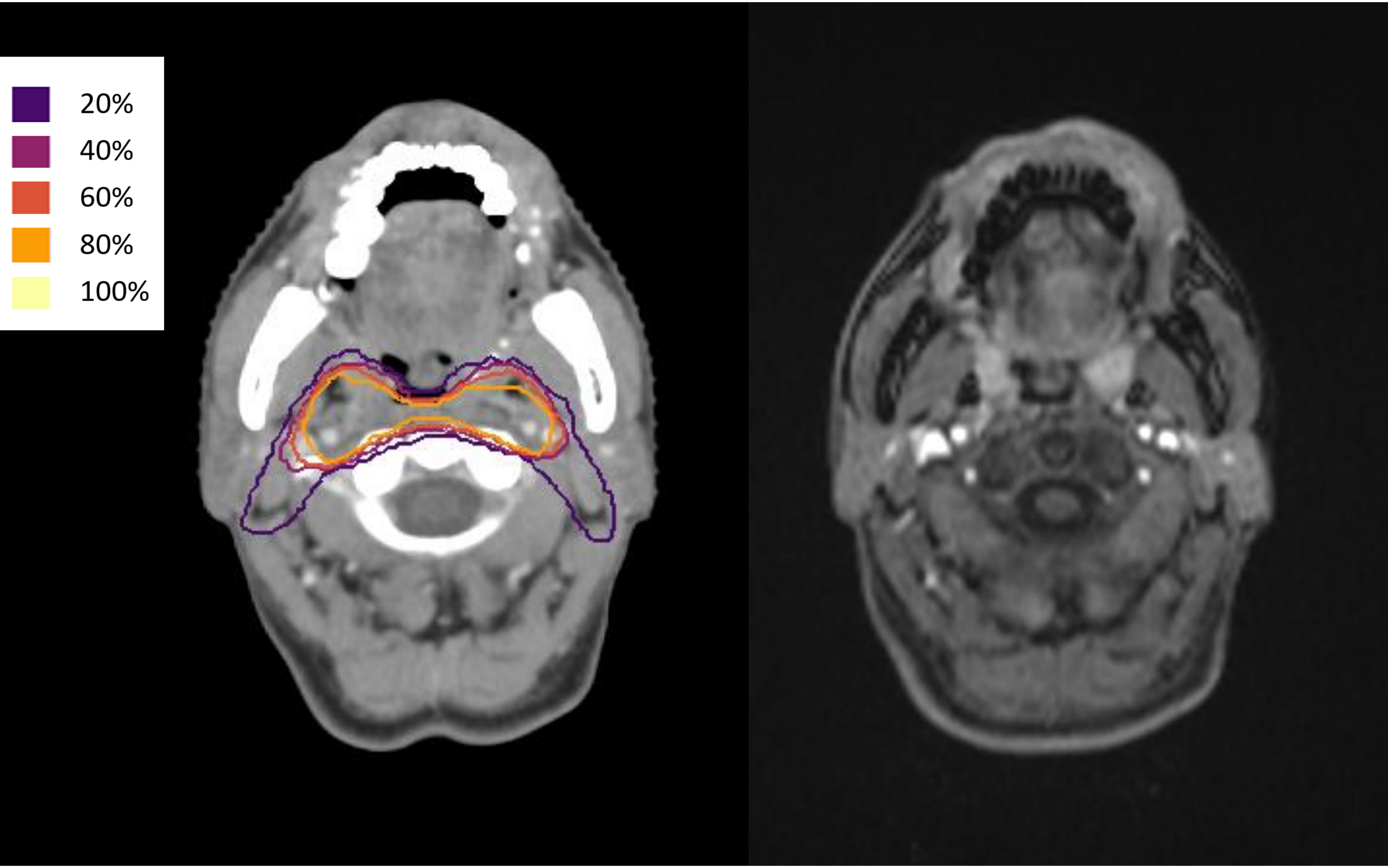
20%
40%
60%
80%
100%

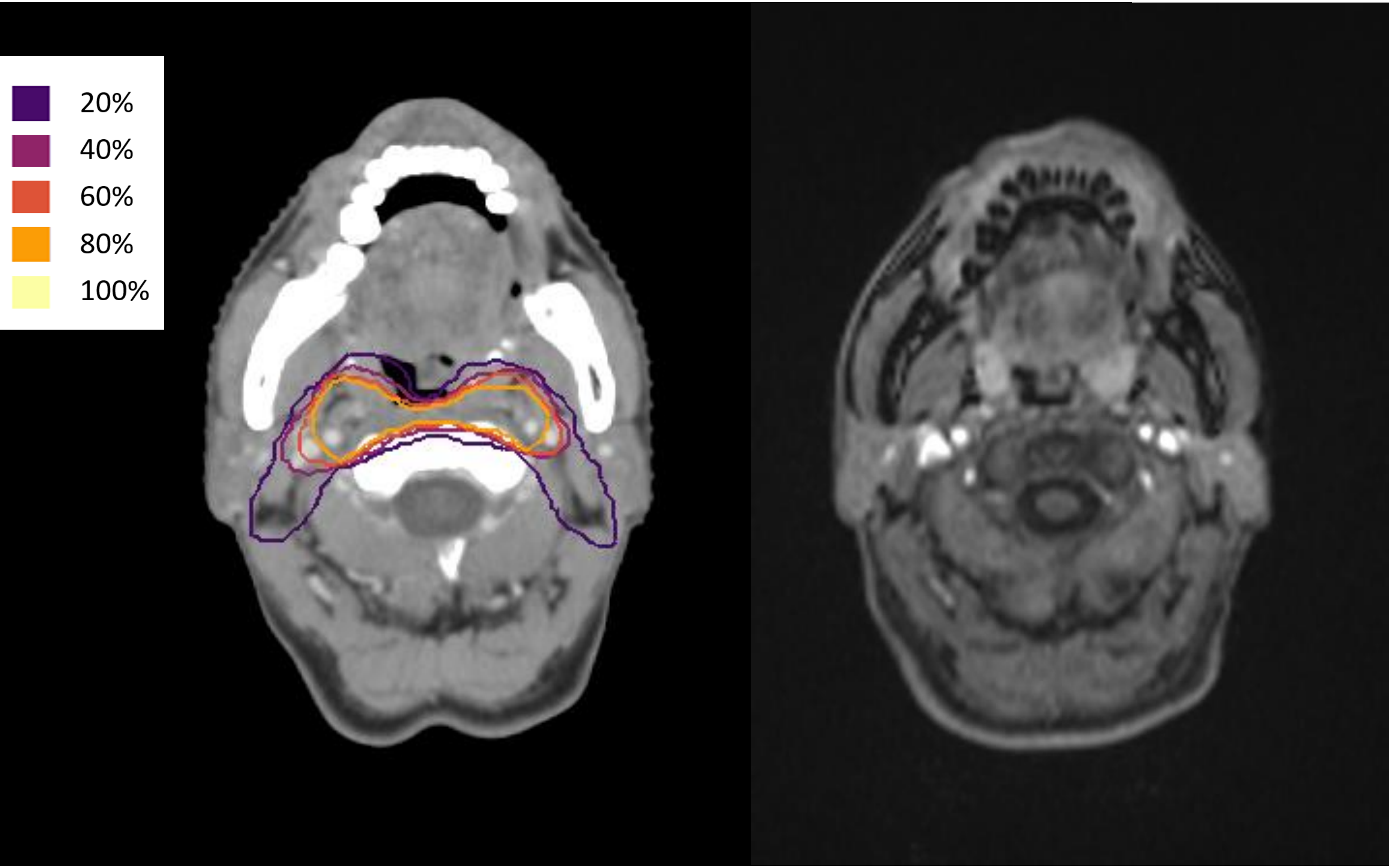
20%
40%
60%
80%
100%

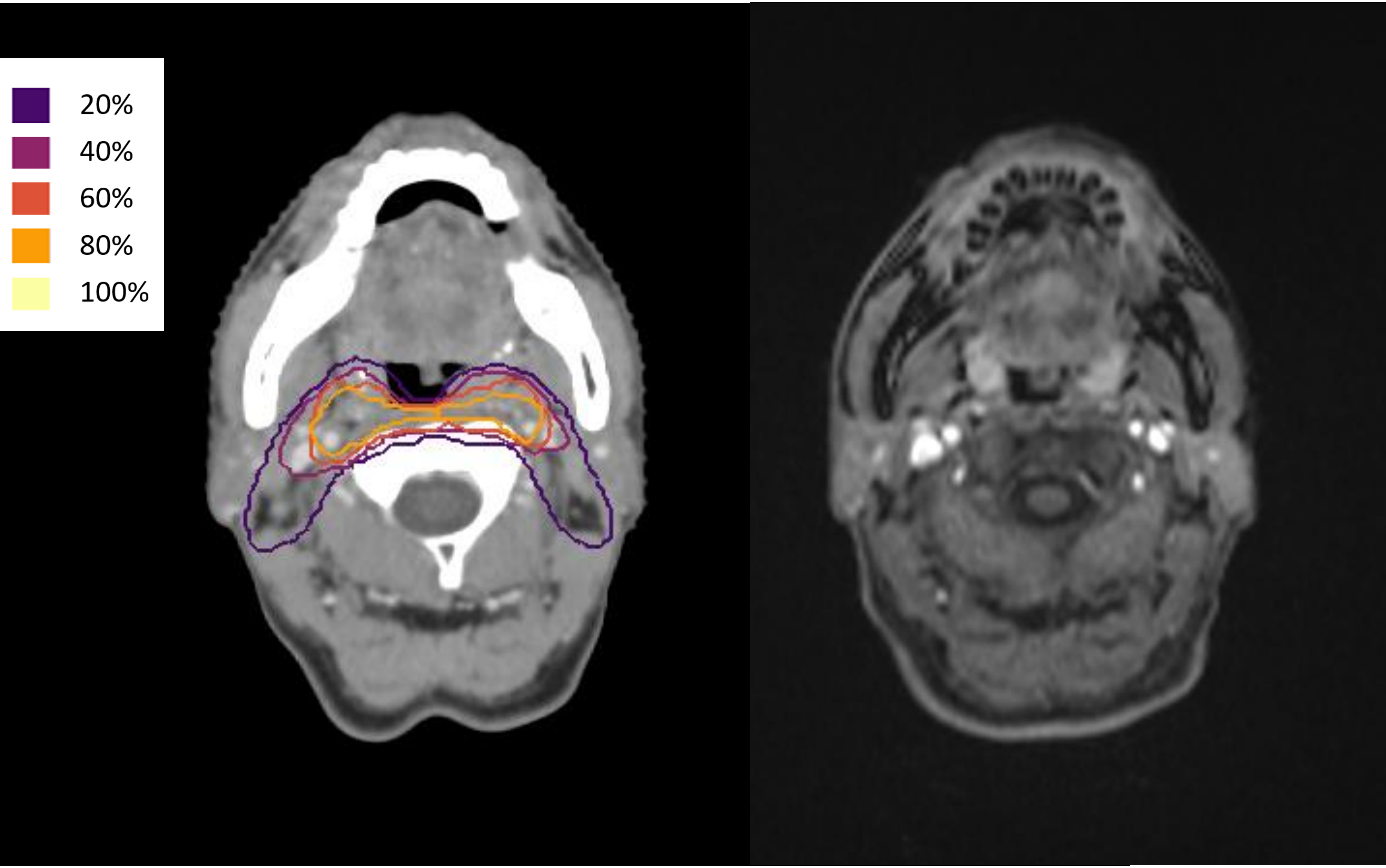
20%
40%
60%
80%
100%

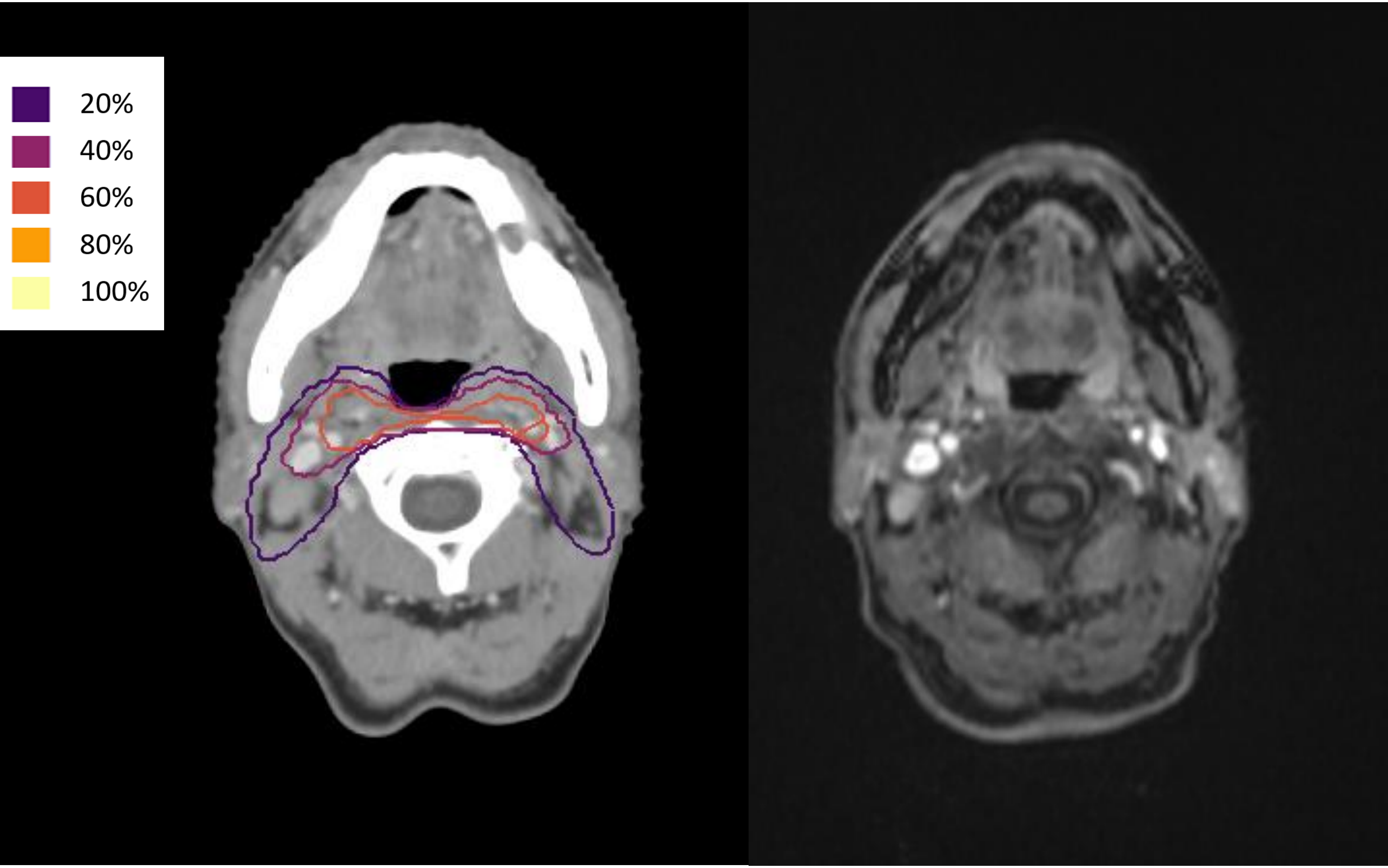
20%
40%
60%
80%
100%

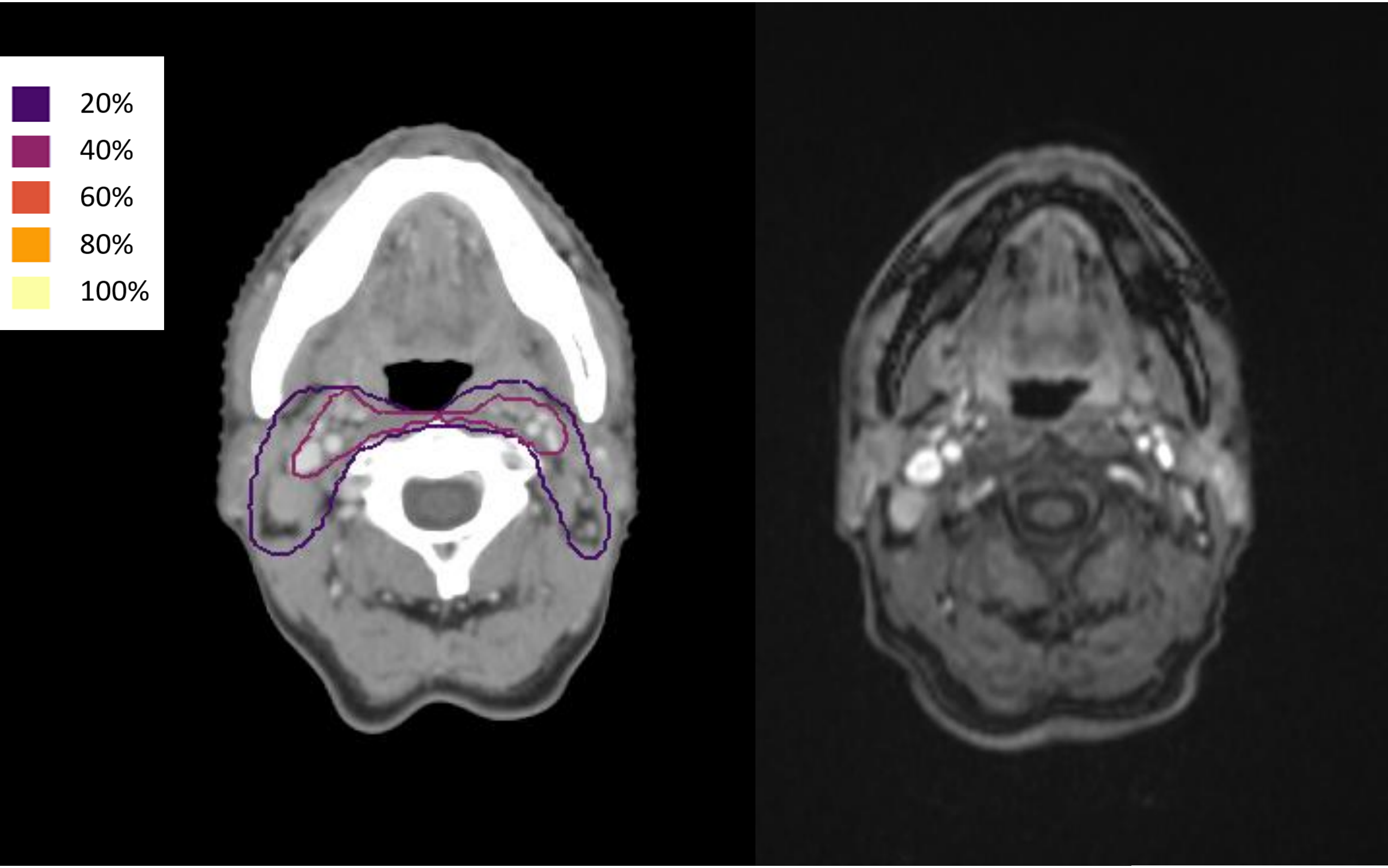
20%
40%
60%
80%
100%

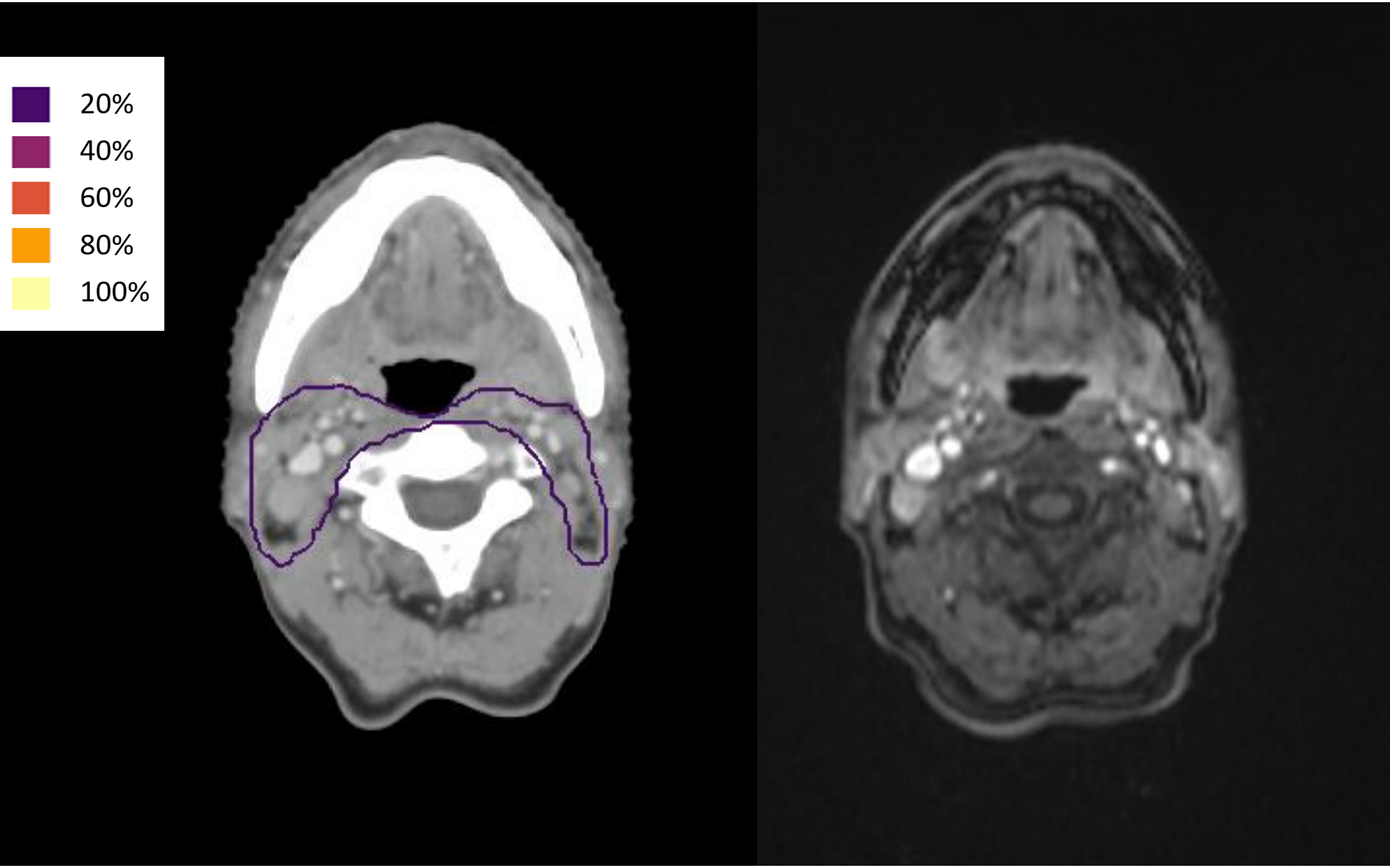
20%
40%
60%
80%
100%
